# AAV-HBV mouse model replicates the intrahepatic immune landscape of chronic HBV patients at single-cell level

**DOI:** 10.3389/fimmu.2025.1421712

**Published:** 2025-06-18

**Authors:** Nádia Conceição-Neto, Wim Pierson, Qinglin Han, Zhiyuan Yao, Qun Wu, Koen Dockx, Liese Aerts, Dries De Maeyer, Matthias Beyens, Koen Van den Berge, Chris Li, George Kukolj, Ren Zhu, Vinod Krishna, Ondřej Podlaha, Isabel Nájera, Ellen Van Gulck

**Affiliations:** ^1^ ID Discovery, Infectious Diseases Therapeutic Area, Johnson & Johnson Innovative Medicine, Beerse, Belgium; ^2^ ID Discovery, Infectious Diseases Therapeutic Area, Johnson & Johnson Innovative Medicine, Shanghai, China; ^3^ ID Discovery, Infectious Diseases Therapeutic Area, Johnson & Johnson Innovative Medicine, Brisbane, CA, United States; ^4^ Charles River Laboratories, Beerse, Belgium; ^5^ Discovery Therapeutics and Molecular Pharmacology, Johnson & Johnson Innovative Medicine, Beerse, Belgium; ^6^ Statistics and Decision Sciences, Johnson & Johnson Innovative Medicine, Beerse, Belgium; ^7^ ID Discovery, Infectious Diseases Therapeutic Area, Johnson & Johnson Innovative Medicine, Spring House, PA, United States

**Keywords:** hepatitis B virus, single-cell transcriptome, AAV-HBV-infected mouse model, T cell exhaustion, chronic HBV

## Abstract

**Introduction:**

Unresolved hepatitis B virus (HBV) infection leads to a progressive state of HBV-specific immune dysfunctionality that characterizes chronic infection. The immune-competent adeno associated virus (AAV)-HBV mouse model is commonly used preclinically, though a comprehensive characterization of the liver immune microenvironment and its translatability to human infection is still lacking. We investigated the intrahepatic immune profile of the AAV-HBV mouse model at a single-cell level and compared with data from CHB patients in immune tolerant (IT) and immune active (IA) clinical stages.

**Methods:**

Immune exhaustion was profiled through an iterative subclustering approach for cell-typing analyses of single-cell RNA-sequencing data in CHB donors and compared to the AAV-HBV mouse model 4-weeks and 24-weeks post-transduction to assess its translatability. This was confirmed using an exhaustion flow cytometry panel at 4 and 42-weeks post-transduction.

**Results:**

Using single-cell RNA-sequencing, CD8 pre-exhausted T-cells with self-renewing capacity (*TCF7+*), and terminally exhausted CD8 T-cells (*TCF7-*) were detected in the AAV-HBV model. These terminally exhausted CD8 T-cells (expressing *Pdcd1, Tox, Lag3, Tigit*) were significantly enriched versus control mice and independently identified through flow cytometry. Importantly, comparison to CHB human data showed a similar exhausted CD8 T-cell population in IT and IA donors, but not in uninfected individuals.

**Discussion:**

Long term high titer AAV-HBV mouse liver transduction led to T-cell exhaustion, as evidenced by expression of conventional immune checkpoint markers at mRNA and protein levels. In both IT and IA donors, a similar CD8 exhausted T-cell population was identified, with increased frequency observed in IA donors. These data support the use of the AAV-HBV mouse model to study classical T-cell exhaustion in HBV infection and the effect of immune-based therapeutic interventions.

## Graphical Abstract

GRAPHICAL ABSTRACT

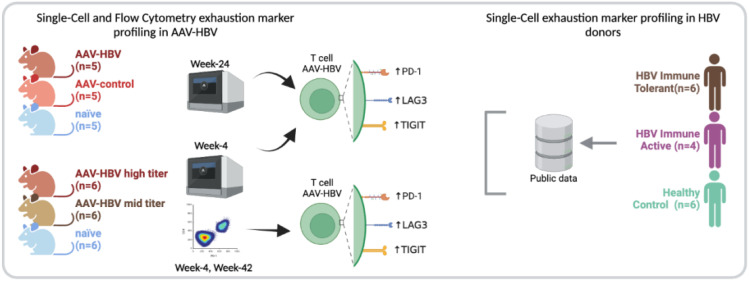

## Highlights

- Mice transduced with high-titer AAV-HBV showed the presence of CD8 exhausted T-cell gene expression markers after 4 weeks, which increased after 24 weeks.
- A similar CD8 T-cell population expressing exhaustion markers identified by single-cell RNA sequencing could be detected through flow cytometry in AAV-HBV-transduced mice after 42 weeks but at a lower frequency after 4 weeks.
- A CD8 T-cell population expressing similar exhaustion markers could be found in chronic HBV donors, but not in healthy individuals.

## Introduction

Chronic infection with the hepatitis B virus (HBV; CHB) affects an estimated 296 million people worldwide, according to the World Health Organization (WHO) (1). HBV is a partially double-stranded DNA virus from the *Hepadnaviridae* family (2) and primarily targets hepatocytes. While the virus is not directly pathogenic, resulting immune responses lead to chronic liver inflammation, which then leads to liver cirrhosis and sometimes hepatocellular carcinoma (3). Despite the availability of effective prophylactic vaccines, vertical transmission still occurs, which leads to chronic infection in >90% of cases (1). For those chronically infected, antiviral therapies based on nucleoside/nucleotide analogs (NAs) control viral replication but generally do not eradicate the virus even after many years. The goal of novel therapies is to bring about a functional cure, a state achieved by those recovering from acute infection, where the immune system appears to completely control viral replication even though the virus is probably never eradicated. With current approaches of stopping NA therapy in those who are eligible, functional cure is declared with at least 24 weeks of stable loss of detectable HBV DNA and surface antigen (HBsAg) after treatment cessation since that duration predicts low relapse rates (2). Only a small fraction of patients achieve functional cure after NA treatment is stopped, with uncured patients likely due to inadequate immune control (4–6). Although the exact mechanisms underlying the impaired immune response are not fully understood, evidence of loss of CD4 T-cell help and dysfunctional HBV-specific CD8 T-cell responses have been reported (7, 8)

During acute viral infections, T-cell responses are believed to follow a pattern of expansion, contraction, memory cell formation, and maintenance. However, in chronic infection, continuous antigen exposure prevents proliferation-competent T cells from returning to a memory quiescent state, which leads to an exhausted T-cell phenotype (9–12)

Exhausted T cells were first described in lymphocytic choriomeningitis virus (LCMV) infection, which was a well-established mouse model of chronic infection. These exhausted T cells were characterized by the upregulation of several inhibitory receptors (such as PD-1, LAG-3, and TIM-3), reduced cytokine production, and diminished cytotoxic activity (13). In chronic HBV, several studies have shown the presence of exhausted and dysfunctional T cells (14–20). The HBV-specific CD8 T cells from chronically infected HBV patients, just like in cancer and other chronic viral infections, are not a functionally homogeneous population of exhausted T cells but are composed of distinct T-cell subsets with different degrees of dysfunction (21, 22) and with different HBV antigen specificities (23). Recent evidence points to a great degree of variability in the level and nature of HBV-specific T-cell dysfunction, including the expression of inhibitory receptors, transcription factors, and metabolic markers, suggesting the HBV-specific T-cell dysfunction beyond classical exhaustion (24). HBV-specific T-cell dysfunction is a distinct immunological state, shaped by weak priming, continuous antigen presence, and the absence of innate activation, which is different compared to the dysfunction of T cells seen in other chronic infections or cancers (25, 26).

The adeno-associated virus (AAV)–HBV mouse model has emerged as an important tool for studying HBV pathogenesis and as a preclinical model for drug discovery (27–29). AAV-HBV transduction can lead to a long-term expression of viral antigens. However, there is an important difference between this model and natural HBV infection since natural HBV infection is characterized by a lack of innate immune activation, leading to defective T-cell priming and atypical exhaustion (26). In contrast, the AAV-HBV model activates innate immunity, which artificially enhances T-cell priming and supports classical exhaustion mechanisms. This makes the AAV-HBV model suitable for studying antigen-driven dysfunction, but not for capturing the full immune evasion strategy of HBV. We aimed to understand whether long-term AAV-HBV transduction in mice would lead to the upregulation of T-cell exhaustion markers. Moreover, we analyzed publicly available data from human chronic HBV donors [immune-tolerant (IT) and immune-active (IA) phases] (30) to assess the similarity between the observed HBV T-cell exhaustion generated in the AAV-HBV mouse model and human CHB infection.

## Materials and methods

### Ethical statement and animal experimentation

Animal experiments were first reviewed, approved, and then conducted in strict accordance with guidelines established by the Janssen Pharmaceutica NV Institutional Animal Care and Use Committee. The local Johnson & Johnson Ethical Committee approved all experimental protocols performed at Janssen. Experiments were performed following the guidelines of the European Community Council directive of November 24, 1986 (Declaration of Helsinki 86/609/EEC). The criteria of the American Chemical Society’s ethical guidelines for the publication of research (31) were met. Every effort was made to minimize animal discomfort and to limit the number of animals used. Mice were kept in a specific pathogen-free facility under appropriate biosafety levels following institutional guidelines.

### Study overview

Male C57BL/6 mice (4–5 weeks old, WuXi AppTec, Shanghai, China) were transduced via the tail vein with either 1 × 10^11^ viral genome equivalents (vge) per mouse of rAAV8-HBV1.3 (high dose, n = 5) (FivePlus Molecular Medicine Institute, Beijing, China) or rAAV vector control (n = 5) or kept naïve (n = 5).

Female C57BL/6 mice (6–8 weeks old, Janvier Labs, Le Genest-Saint-Isle, France) were transduced with rAAV8-HBV1.3 (BrainVTA, Beijing, China) at 3 × 10^10^ vge/mouse (high dose, n = 6 or n = 7) or 2.5 × 10^9^ vge/mouse (mid dose, n = 6 or n = 7) via the tail vein or kept naïve (n = 6). Twenty days after transduction, mice were randomized based on serum HBsAg levels. Four (n = 6/dose group) and 42 weeks (n = 7/dose group) post-transduction, mouse liver samples were collected.

Four weeks post-transduction, mouse liver samples were collected for single-cell RNA sequencing and flow cytometry. Twenty-four weeks post-transduction, mouse liver samples were collected for single-cell RNA sequencing, and 42 weeks post-transduction, mouse liver samples were collected for the evaluation of protein expression through flow cytometry.

At regular time points post-transduction, blood for viral parameters was collected via the saphenous vein, from which serum was prepared, and stored at −80°C until being assayed.

### Viruses

For the rAAV-HBV1.3 vector construction, genotype D was used, and the construct reference can be found on National Center for Biotechnology Information (NCBI) under accession number KX470733. Control vector and naïve mice were randomly selected to provide an equivalent body weight distribution with the AAV-HBV mice.

rAAV vector control contained an empty rAAV capsid and rAAV containing a short sequence of the CMV promoter and BGH poly A tail flanked with inverted AAV-ITR.

### Viral parameters and alanine aminotransferase analyses

Serum HBsAg and hepatitis B e-antigen (HBeAg) were quantified using CLIA kits (Cat. No. CL18002, CL18005, Ig Technology, Burlingame, CA, USA), which were used according to the manufacturer’s guidelines. Dilutions of serum in phosphate-buffered saline (PBS) were used to ensure the assay sensitivity of the viral markers in the linear range of the assy. Read-out of plates was performed using a Viewlux Ultra HTS microplate imager (PerkinElmer, Mechelen, Belgium).

### Mouse liver intrahepatic immune cell isolation for single-cell RNA-sequencing at 24 weeks

Intrahepatic immune cells (IHICs) were obtained by perfusing the whole liver with PBS via the hepatic portal vein to prevent contamination from circulating blood; single-cell suspensions of mouse IHICs were acquired via a mechanical dissection method using the gentleMACS (Miltenyi Biotec, Bergisch Gladbach, Germany). Homogenized liver tissue was centrifuged at 50 × *g* for 1 minute to decrease hepatocyte contamination. Cell suspensions were further purified with 35% Percoll (GE, 17-0891-09) in PBS and underwent density gradient centrifugation at 800 × *g* for 20 minutes, followed by RBC lysis (004333-57; eBioscience, San Diego, CA, USA). Cell concentration and viability were then determined using a Countess^®^ II Automated Cell Counter (Invitrogen, Carlsbad, CA, USA).A total of 20,000 cells were used to perform single-cell RNA sequencing.

### Mouse liver IHIC isolation for flow cytometry and single-cell RNA-sequencing at 4 weeks and for flow cytometry at 42 weeks

IHICs were obtained by perfusing the liver with PBS via the hepatic portal vein to prevent contamination from circulating blood; tissue disruption was performed using a gentleMACS dissociator and the enzymatic liver dissociation kit for mice, according to the manufacturer’s protocol. Hepatocytes were separated from lymphocytes by centrifugation at 50 ×*g* for 5 minutes. The supernatant was centrifuged at 400 ×*g* for 5 minutes followed by resuspension in an isotonic 33.75% (*v*/*v*) Percoll (GE Healthcare, Chicago, IL, USA) diluted in PBS with 2% fetal calf serum and underwent density gradient centrifugation at 700 × *g* for 12 minutes. Next, residual hepatocytes and debris were discarded, and red blood cells co-sedimented with the IHICs were lysed using an ACK lysing buffer (Lonza, Basel, Switzerland) for 5 minutes. Cells were washed twice and counted. Cell concentration and viability were then determined using a Nexcelom Cellaca MX Cell Counter (PerkinElmer). For mice transduced for 4 weeks with AAV-HBV, 1 million cells were used to perform flow cytometry staining. A total of 20,000 cells were used to perform single-cell RNA sequencing. For mice transduced for 42 weeks with AAV-HBV, 1 million cells were used to perform flow cytometry staining.

To retrieve Kupffer cells and liver sinusoidal endothelial cells (LSECs), an enzymatic digestion was performed for this study, instead of a mechanical digestion, as performed for the single-cell RNA-sequencing study at 24 weeks.

### Single-cell RNA sequencing and data analysis

IHICs were processed according to the 10x Chromium Single-cell V(D)J Reagent Kits User Guide and loaded onto the Chromium 10x platform using the 5′ v1.1 chemistry (10x Genomics, Pleasanton, CA, USA). Libraries were sequenced on a NovaSeq 6000 platform (PE150) (Illumina) to an average of ~50,000 reads per cell. Read alignment was conducted using the Cell Ranger pipeline (version 7) against the mouse genome reference (mm10). Resultant cell-by-gene matrices for each sample were merged per time point (due to protocol isolation differences) across all conditions tested and samples. Pre-processing, alignment, and data filtering were applied equivalently to all samples using our internal pipelines based on OpenPipeline. Cells with less than 1,000 Unique Molecular Identifiers (UMIs), less than 200 genes, or more than 25% mitochondrial counts were removed from downstream analysis. All downstream analyses were conducted in R v4.0.5 (58) using the Seurat v4.1.1 package (59).

Data were log-normalized with a scaling factor of 10,000. The top 2,000 most variable genes as determined by the “vst” method implemented as the FindVariableFeatures function were selected and scaled using a linear model implemented as the ScaleData function. Afterward, principal component analysis (PCA) was run, and the number of significant principal components (PCs) to be used for downstream cell clustering was determined using an ElbowPlot and heatmap inspection. A nearest neighbor graph and a Uniform Manifold Approximation and Projection (UMAP) plot were generated using the significant PCs.

A Louvain clustering was run on all cells, and the best resolution for clustering was determined using an average silhouette scoring across all clusters, testing 10 resolutions between 0.1 and 1 as previously implemented in Ziegler et al. (60). Marker genes for each cluster were calculated using the FindAllMarkers function (method=“wilcox”), and each cluster was iteratively subclustered further using the same approach. Subclustering was stopped when the resulting clusters were not meaningfully different and no significant marker genes could be identified. Clusters were annotated as cell-type populations based on the expression of their respective marker genes.

### Single-cell RNA-sequencing data differential expression analysis

A subpopulation pseudo-bulk analysis was performed using muscat v1.4.0 (61). Each cell subpopulation’s raw data counts were aggregated to pseudo-bulk data using the aggregateData function with the fun=“sum” option. The differential state was assessed using the pbDS function with the following parameters: method=“edgeR” and min_cells=1 (edgeR v3.32.1 (62)). Enrichment analysis was performed using the Gene Set Enrichment analysis of Gene Ontology (gseGO) function in the clusterProfiler v4.3 R package (63) [with a false discovery rate (fdr) adjusted p-value <0.05].

### Single-cell RNA-sequencing data receptor–ligand analysis at 4 and 24 weeks

After cell annotation, CellChat v1.1.3 (33) implementation in R v4.0.5 (58) was used to construct a cell–cell communication network per condition. For naïve, AAV-control, and AAV-HBV samples, a network based on known ligand–receptor pairs and ligands was calculated using the standard analysis, omitting population size to decrease bias against low abundant cell populations. A total of 33 immune cell subtypes (all) were used for analysis, resulting in 1,089 source-target cell population pairs. To narrow down the list, the top 1% of cell population pairs that showed the biggest differences between the AAV-HBV and naïve groups within the CD8 exhausted T cells (z-score) were selected.

### Human CHB dataset

Raw fastq files from the human chronic HBV dataset of Zhang et al. (27)were downloaded from Gene Expression Omnibus (GSE182159). Read alignment was conducted using Cell Ranger 7.0.0 (64) against the human genome reference (GRCh38). Aligned cell-by-gene matrices for each sample were merged. All downstream analyses were conducted as described above for the mouse samples.

### Flow cytometry

All staining procedures were performed for 30 minutes at 4°C, and all washing steps were conducted at 400 ×*g* for 5 minutes at 4°C. Cells were treated with True stain monocyte blocker (BioLegend, San Diego, CA, USA) and an anti-CD16/CD32 FC blocking reagent (clone 2.4G2, BD Biosciences, San Jose, CA, USA) for 10 minutes, after which a dead cell exclusion marker (fixable viability dye eFluor780, Invitrogen) was co-incubated for 30 minutes. After washing the cells with stain buffer, staining was performed on ice using the following panel of fluorochrome-conjugated antibodies diluted in stain buffer [0.5% bovine serum albumin (BSA) supplemented with brilliant stain buffer plus (BD)]: CD45-BUV395 (clone 30-F11), CD8a-BUV496 (clone 53-6.7), CD4-BV786 (clone GK1.5), CD3-PerCP-Cy5.5 (clone 17A2), CXCR3-BV510 (clone CXCR3-173), PD1-BV605 (clone J43), LAG3-BUV737 (clone C9B7W), and TIM3-BB515 (clone 5D12) (BD) and TIGIT-PE (clone A17200C), CD154-PE-Cy7 (clone MR1), and CD155-BV711 (clone TX56) (all monoclonal antibodies (mAbs) from BioLegend). After staining of cell surface proteins, cells were washed twice with stain buffer, fixed with a fixation reagent (Invitrogen), and permeabilized twice using the Foxp3/Transcription Factor Staining buffer set (Invitrogen) before being stained on ice with a second panel of antibodies added to diluted permeabilization buffer (BD): TOX-A647 (clone NAN448B, BD), Foxp3-PE-CF594 (clone 3G3, BD), and TCF1-A405 (clone #812145, R&D Systems, Minneapolis, MN, USA). Finally, cells were washed twice with permeabilization buffer and left in 200 µL of stain buffer BSA in the dark at 4°C until cytometry data were acquired on a BD LSRFortessa instrument. Data were analyzed using FlowJo v10.8.1 (BD). The gating strategy is shown in **Supplementary Figure S1**.

### Statistical analysis

Statistical comparisons were performed using either GraphPad Prism 9 or package rstatix v0.7.2 (65) in R v4.0.5. Differential abundance in single-cell RNA sequencing was calculated on cell proportions using a Wilcoxon test, and the p-value was adjusted using the Bonferroni correction across all contrasts and populations.To compare statistical differences in protein expression between distinct groups, a Kruskal–Wallis test with Dunn’s correction for multiple comparisons was performed, and significant p-values (p < 0.05) are indicated in the respective figures.

## Results

### Intrahepatic immune cell atlas from AAV-HBV mice vs. control mice

**Figure 1:**
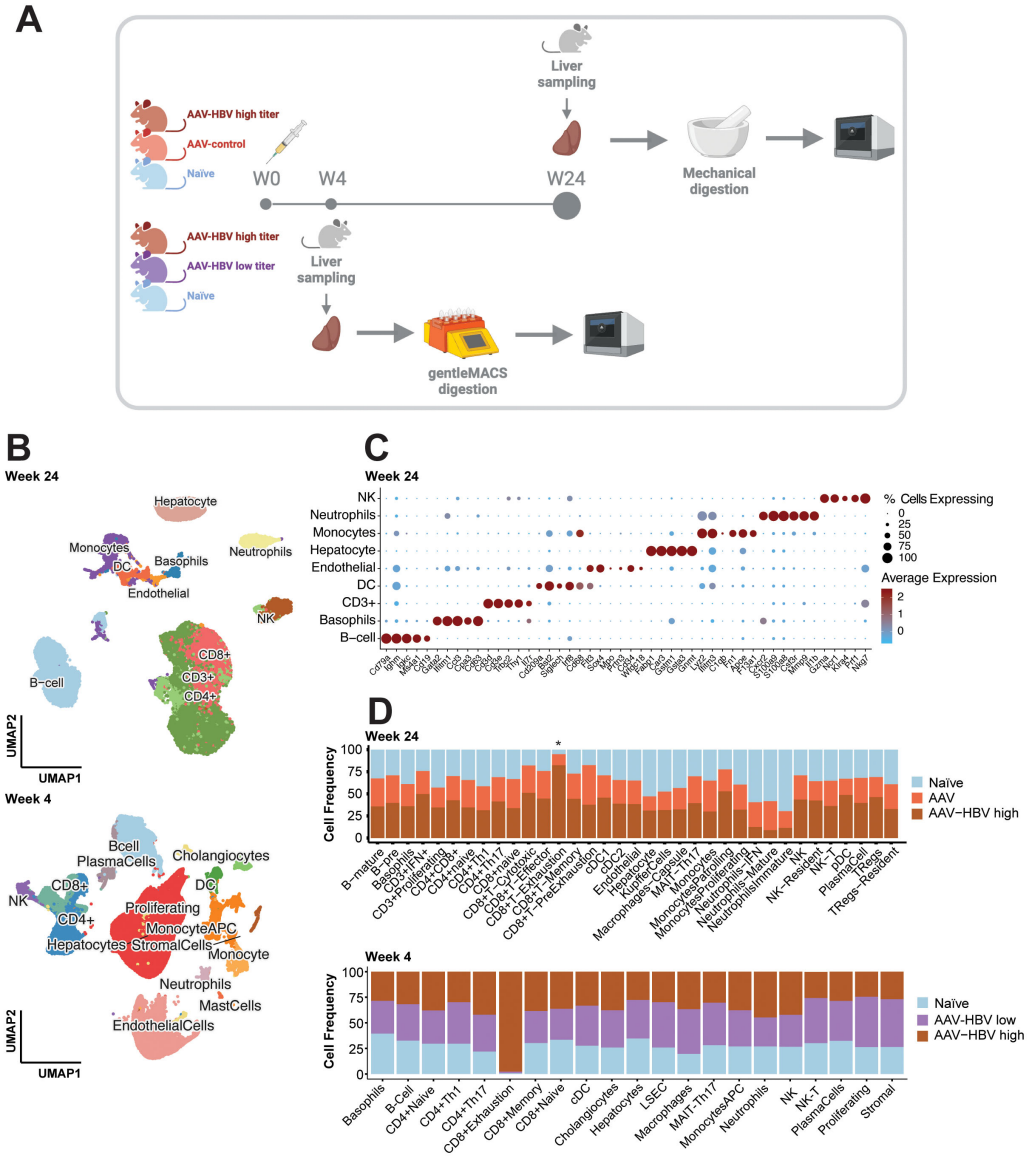
Single-cell RNA-sequencing profiling of the AAV-HBV mouse model. Liver samples from male C57BL/6 mice from three groups were collected: infected with AAV-HBV, infected with AAV-control, and naïve 4 weeks post-transduction. Liver samples from female C57BL/6 mice from three groups were collected: naïve and infected with AAV-HBV high or mid titer 24 weeks post-transduction. **(A)** Graphical overview of the experimental design using liver mechanical digestion for intrahepatic lymphocyte isolation and single-cell RNA-sequencing profiling. **(B)** UMAP projection of the major cell types identified from 15 samples in the 24-week study and 18 samples in the 4-week study. **(C)** Dotplot of scaled data with expression of the top 5 marker genes across each major cell type determined using Wilcoxon rank sum test. **(D)** Cell proportion (%) plot across all cell types (deepest cell annotation) in the different mouse groups. AAV-HBV, adeno-associated virus–hepatitis B virus; UMAP, Uniform Manifold Approximation and Projection.

To build a murine HBV-specific immune cell atlas and characterize the T-cell exhaustion markers after prolonged HBV replication, we first characterized the AAV-HBV mouse model and then compared it to the human liver atlas samples from IT and IA CHB patients (30). Liver samples from mice transduced with high-titer and mid-titer AAV-HBV, to mimic different levels of HBV antigenemia, or naïve mice were collected 4 weeks post-transduction (**Figure 1A**). As a result of a successful AAV-HBV transduction, average HBsAg levels of 4.40 log_10_ IU/mL (± 0.44 log_10_ IU/mL) in high titer and 3.18 log_10_ IU/mL (± 0.15 log_10_ IU/mL) in mid titer, and 3.44 log_10_ IU/mL (± 0.05 log_10_ IU/mL) and 2.20 log_10_ IU/mL (± 0.10 log_10_ IU/mL) for HBeAg levels, respectively, could be detected in all mice at week 4 (**Supplementary Figures S2A, B**). Liver samples from mice transduced with high-titer AAV-HBV, AAV-control, or naïve mice were collected 24 weeks post-transduction. At week 24, average HBsAg levels of 4.56 log_10_ IU/mL (± 0.09 log_10_ IU/mL) and an average of 2.82 log_10_ IU/mL (± 0.11 log_10_ IU/mL) for HBeAg levels could be detected in all mice transduced with high titer of AAV-HBV (**Supplementary Figures S2C, D**).

Samples across all groups showed good recovery of cells (**Supplementary Figure S3**), capturing a total of 79,970 (24-week study; **Supplementary Figure S3A**) and 198,389 (4-week study; **Supplementary Figure S3B**) high-quality (HQ) mouse immune and non-immune cells. In the week 24 samples, all major immune cell populations (Ts, Bs, NKs, DCs, monocytes, and neutrophils) and hepatocytes (**Figure 1B**, **Supplementary Figure S4A**) were covered. The cell surface markers characteristic for respective cell populations are described in detail in **Supplementary Table S1**. Based on the recovered cells from all samples, we observed that Kupffer cells constituted 0.1% of the total cells and 5% of hepatocytes, whereas LSECs were not detected (using *Clec4g*, *Aqp1*, and *Dnase1l3* as markers). In contrast, T cells (52%) and B cells (22%) were the most prevalent cell types consistently recovered across all groups (**Supplementary Table S2**). All major cell populations were well defined with hallmark genes (**Figure 1C**, **Supplementary Figure S5**) such as *Cd3d* for T cells, *Cd79a* for B cells, *Fapb1* for hepatocytes, *Cst3* for dendritic cells, *Lyz2* for monocytes/macrophages, *Fcerg1* for NK cells, *Cd63* for basophils, *S100a8/9* for neutrophils, and *Prtn3* for endothelial cells (**Supplementary Table S1**). After performing the first-level cell annotation analysis, each cluster was subclustered for more granularity (**Supplementary Figures S4**-**S7**). B cells were further subdivided into plasma cells, pre-B cells, and mature B cells (**Supplementary Table S1**). Within the T-cell compartment, a first subclustering identified T cells positive for *CD8*, *CD4*, and *CD3+*, and each T-cell subtype was further defined based on key marker genes (**Figure 1C**, **Supplementary Figures S5**, **Supplementary Figures S6**, **S8**). The dendritic cells were subdivided into pDCs, cDC1, and cDC2. The monocyte compartment contained classical monocytes, patrolling monocytes, and proliferating monocytes; the macrophages identified were Kupffer cells and capsule macrophages. For the NK cells, a liver-resident NK population was identified. Finally, the neutrophils were subdivided into mature neutrophils, type I interferon neutrophils (neutrophils–IFN), and immature neutrophils.

All cell subpopulations could be identified across the three treatment groups (AAV-HBV high, AAV-control, and naïve mice) in analyses of samples from week 24 post-transduction (**Figure 1D**, **Supplementary Figure S7A**, **Supplementary Table S2**) and week 4 post-transduction (AAV-HBV high, AAV-HBV mid, and naïve mice; **Figure 1D**, **Supplementary Figure S7B**, **Supplementary Table S3**). At week 4, due to the nature of the protocol isolation, the majority of cells detected were hepatocytes (31%) and T cells (21%), followed by LSECs (18%) (**Supplementary Figure S4B**). At week 24, no statistically significant differences in cell subset proportions between AAV-HBV and AAV-control samples were observed at the highest level of cell annotation (**Supplementary Table S4**). A deeper analysis of the T-cell compartment (CD8+, CD4+, and CD3+) between AAV-HBV and AAV-control samples showed no differences among CD4 and CD3 in the presence/absence of HBV replication (**Supplementary Table S5**). However, in the CD8 T-cell subset, there were proportional differences. From all exhausted T cells identified, in the 24-week study, 75% of them were observed in the AAV-HBV arm; in the 4-week study, 98% of the exhausted T cells were identified in the high-titer AAV-HBV mice (**Figure 1D**, **Supplementary Table S5**). At week 4, no statistically significant differences were observed across AAV-HBV high, mid, and naïve mice (**Supplementary Table S6**).

### Prolonged HBV replication induces an expansion of CD8 T cells with an exhaustion phenotype in the AAV-HBV mouse model

**Figure 2:**
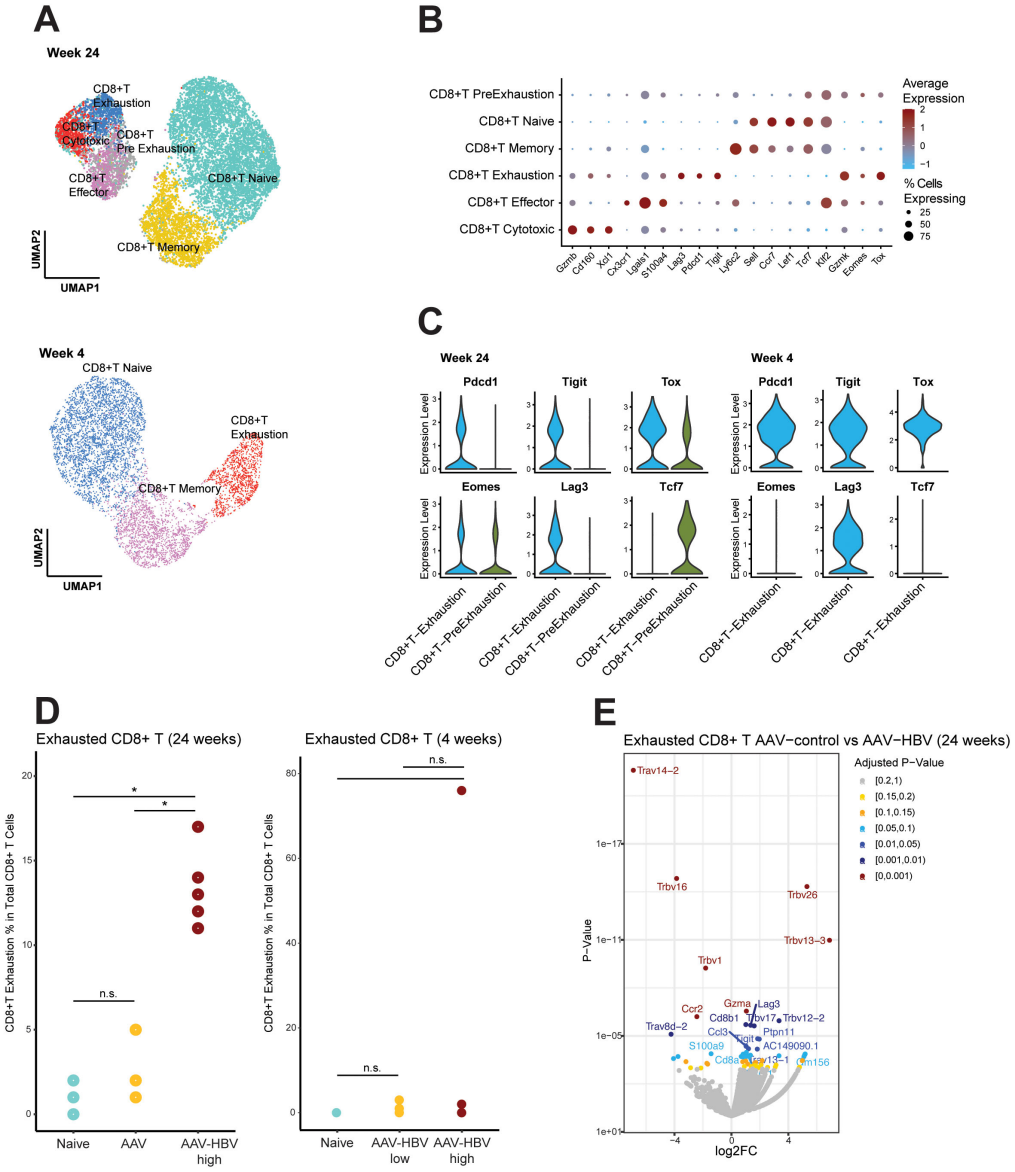
Single-cell RNA sequencing demonstrates CD8 T-cell exhaustion in AAV-HBV mouse model. **(A)** UMAP projection of the deeper annotation of all CD8 T-cell populations across the different mouse groups. **(B)** Dotplot with expression of the top 3 marker genes across each deepest CD8 T-cell annotation. **(C)** Violin plots of log normalized data from the exhaustion markers *Pdcd1*, *Tigit*, *Tox*, *Eomes*, *Lag3*, and *Tcf7* across the exhausted T cells and pre-exhausted T cells. **(D)** Frequency of the CD8 exhausted T cells across the different mouse groups. Statistical comparisons were conducted using a non-parametric Wilcoxon test and Bonferroni correction. Comparisons with adjusted p-value <0.05 are shown with (*). **(E)** Volcano plot showing the differentially expressed genes calculated using edgeR between AAV-control and AAV-HBV in CD8 exhausted T cells. Positive log_2_ fold-changes indicate higher expression in AAV-HBV. Genes are colored by adjusted p-value. AAV-HBV, adeno-associated virus–hepatitis B virus; UMAP, Uniform Manifold Approximation and Projection.

The CD8 T-cell compartment showed a broad range of cell types after 4 weeks, but especially after 24 weeks of AAV-HBV transduction and HBV replication (**Figures 2A, B**), from naïve to more effector states across HBV relative to the control arms. In the AAV-HBV mice transduced with AAV-HBV for 24 weeks, CD8 naïve T cells expressed high levels of *Tcf7*, *Sell*, and *Lef1*; CD8 naïve to memory cells also expressed the same naïve markers, but at lower levels, and high levels of *Ly6c2* (32). The cytotoxic CD8 T cells expressed granzyme B (*Gzmb*), *Cd160*, and *Xcl1* (33). The effector CD8 T cells expressed the well-described *Cx3cr1* (34). Interestingly, two sub-populations, labeled pre-exhausted T cells (Tpex) and exhausted T cells (Tex), were identified.

The Tpex population (**Figure 2C**) expressed checkpoint inhibitor markers (*Tox* and *Eomes*) and *Tcf7* (encoding for TCF-1, which is lost upon transition to a fully exhaustion phenotype (35)). However, the Tex expressed *Pdcd1* (encoding PD-1), *Tigit*, *Tox*, *Eomes*, and *Lag3*, but not *Tcf7* (**Figure 2C**). When testing for differential abundance on the relative abundance of the Tex population, both AAV-control (Wilcoxon rank-sum test, p-value = 0.036) and naïve (Wilcoxon rank-sum test, p-value = 0.033) mouse groups differed significantly from AAV-HBV mice, but there was no difference in the Tex population between AAV-control and naïve mice (Wilcoxon rank-sum test, p-value = 0.525) (**Figure 2D**). This means that they both had no or very low levels of Tex compared to the AAV-HBV arm. The abundance of Tpex did not significantly differ across any of the groups (**Supplementary Figure S9**).

More than 10% (mean 13.4%, SD ± 2.3) of total CD8 T cells from AAV-HBV mice were characterized as Tex in the HBV-AAV arm, whereas most CD8 T cells in the control arms (both AAV-control and naïve mice) were phenotypically functional, as Tex were significantly less prevalent with a mean of 2.2% (SD ± 1.6) and 1.0% (SD ± 0.7) of total CD8 T-cell population, respectively.

After 4 weeks of AAV-HBV transduction, no pre-exhausted T-cell population was observed in mice, and terminally exhausted T cells could be identified in one mouse, expressing *Lag3*, *Tigit*, *Tox*, and *Pdcd1*, but not *Eomes* (**Figure 2C**). Therefore, the frequency of exhausted CD8 T cells was mainly driven by one mouse in the high-titer HBV group but otherwise not significantly different from naïve mice independent of HBsAg levels (**Figure 2D**).

Differential expression analysis within the Tex between AAV-control and AAV-HBV in the 24-week study showed significantly increased *Tigit*, *Lag3*, and *Gzma* expression in the AAV-HBV arm relative to the AAV-control arm, indicating that the exhaustion phenotype was associated with the transduced HBV and not the AAV vector (**Figure 2E**).

### CD8 T-cell phenotypic exhaustion

First, two independent flow cytometry studies were performed to confirm the single-cell RNA-sequencing findings, demonstrating less exhausted T cells in the AAV-HBV mouse model 4 weeks post-transduction and T-cell exhaustion in the AAV-HBV mouse model 42 weeks post-transduction. Second, flow cytometry analyses were also used to assess whether T-cell exhaustion in the AAV-HBV mouse model at 42 weeks was influenced by the level of HBV antigenemia. C57BL/6 mice were transduced with either a high titer of AAV-HBV (3 × 10^10^ vge/mouse) or a mid titer of AAV-HBV (2.5 × 10^9^ vge/mouse). By week 4, the high-titer mice reached average HBsAg levels of 4 log_10_ and 3 log_10_ IU/mL, which were maintained at a steady state until the end of the 42-week study (**Supplementary Figures S2E, F**). IHICs were stained for immunophenotyping through flow cytometry using relevant markers identified by single-cell RNA sequencing: PD-1, LAG-3, TIGIT, TOX, and TCF-1. These markers were supplemented with TIM-3, a well-known checkpoint inhibitor marker, even though gene *Havcr2* was not detected in the AAV-HBV exhausted population by single-cell RNA (scRNA) sequencing.

**Figure 3:**
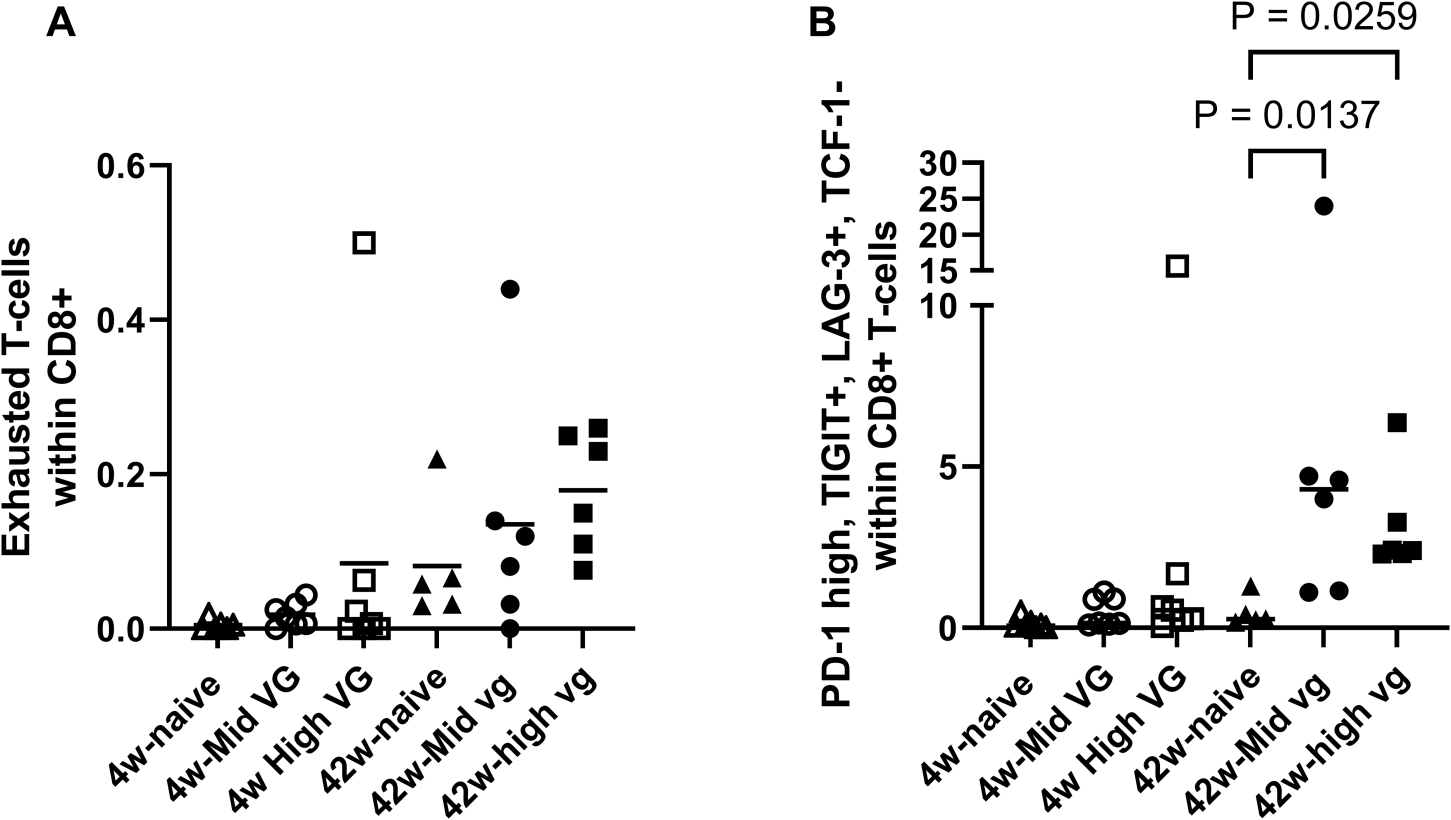
Flow cytometry demonstrated CD8 T-cell exhaustion in AAV-HBV mouse model (female). **(A)** Percentage of exhausted T cells (defined as PD-1+, TIGIT+, LAG-3+, TOX+, and TCF-1−) present in liver in CD8 T-cell population. **(B)** Percentage of PD-1+, TIGIT+, LAG-3+, and TCF-1− T cells in the CD8 T-cell population of the liver. A Kruskal–Wallis test (Dunn’s multiple-comparison test) was performed across the groups, and significant comparisons are highlighted. p-Values are significant if p < 0.05. AAV-HBV, adeno-associated virus–hepatitis B virus.

An initial flow cytometry analysis was performed to compare the frequency of exhausted CD8+ T cells, defined as PD-1+, TIGIT+, LAG-3+, TOX+, and TCF-1−, between naïve and AAV-HBV-transduced mice (**Figure 3A**). Reminiscent of the single-cell RNA sequencing analyses at 4 weeks post-transduction of AAV-HBV, the frequency of exhausted T cells was very low, and there was no statistical difference in immunophenotyping frequency of exhausted CD8 T cells between naïve mice and AAV-HBV-transduced mice (**Figure 3A**). In the study where mice were transduced for 42 weeks, the naïve mice had a slightly higher frequency of exhausted CD8 T cells (0.08%) compared to the naïve mice (0.005%) in the 4-week study, which reflects a known characteristic of aging mice (36). Furthermore, in mice that were transduced with AAV-HBV for 42 weeks, there was a trend for increased frequency of exhausted CD8 T cells (0.08% in naïve mice in contrast to 0.18 in high-titer AAV-HBV mice, p = 0.08). The trend with exhausted CD8 T cells in the liver was more apparent with the high titer of AAV-HBV used to transduce the mice (0.14% in mid titer compared to 0.18% in high titer; not significant, p = 0.82).

When exhausted T cells are defined as PD-1+, LAG-3+, TIGIT+, and TCF-1−, the frequency in the 42-week AAV-HBV-transduced mice is significantly higher compared to that in naïve mice (p = 0.0137 for mid titer and p = 0.0259 for high titer; **Figure 3B**). These results are consistent with the single-cell RNA sequencing data in AAV-HBV-transduced mice showing a higher frequency of CD8 T-cell exhaustion over time.

**Figure 4:**
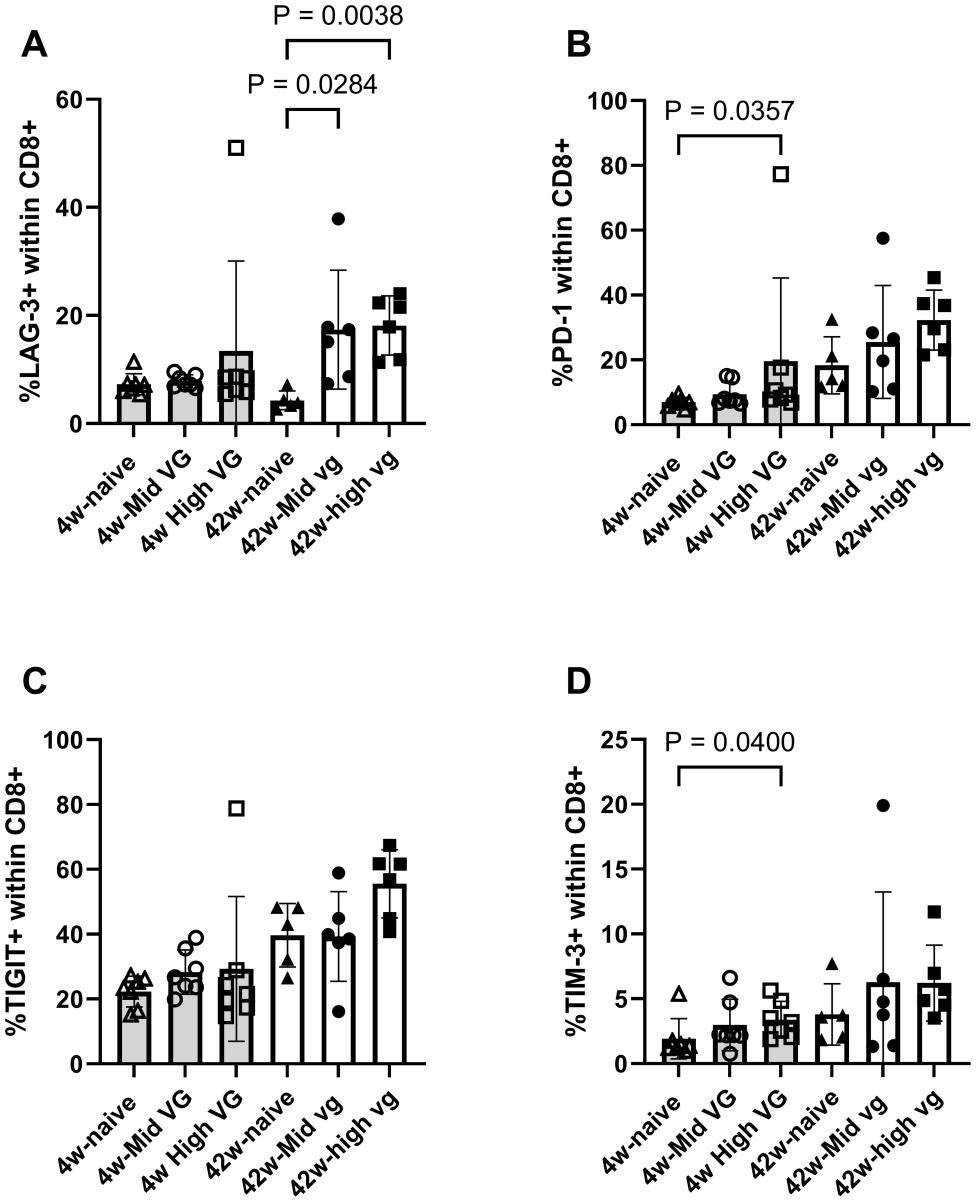
Flow cytometry demonstrates exhaustion marker expression on liver CD8 T cells isolated from female AAV-HBV-transduced mice. **(A)** Percentage LAG-3+, **(B)** PD-1+, **(C)** TIGIT+, and **(D)** TIM-3+ CD8 T cells. Data are shown as mean with errors (SD) from a single experiment run. Kruskal–Wallis test (Dunn’s multiple-comparison test) was performed across the three groups of each study, and significant comparisons are highlighted. AAV-HBV, adeno-associated virus–hepatitis B virus.

Immunophenotyping of checkpoint receptors on CD8 T cells through flow cytometry analyses was also assessed relative to the level of HBV antigenemia (**Figures 4A–D**). Among the mice that were transduced with high-titer AAV-HBV for 4 weeks, there was a significant increase in the proportion of CD8 T cells expressing PD-1 (p = 0.0357; **Figure 4B**) and TIM-3 (p = 0.04; **Figure 4D**), as compared to the naïve mice. The prevalence of LAG-3+ and TIGIT+ CD8 T cells was comparable with that of naïve mice. In mice that were transduced with AAV-HBV for 42 weeks, the proportion of LAG-3+ CD8 T cells was significantly greater in mid-titer and high-titer mice (p = 0.0284 and p = 0.0038, respectively; **Figure 4A**). The frequency of CD8 T cells expressing PD-1 (**Figure 4B**), TIGIT (**Figure 4C**), and TIM-3 (**Figure 4D**) was not significantly different between the different groups. Relative to mid-titer AAV-HBV-transduced mice, there was a trend for a higher frequency of PD-1+ and TIGIT+ CD8 T cells in the high-titer AAV-HBV-transduced mice at 42 weeks.

**Figure 5:**
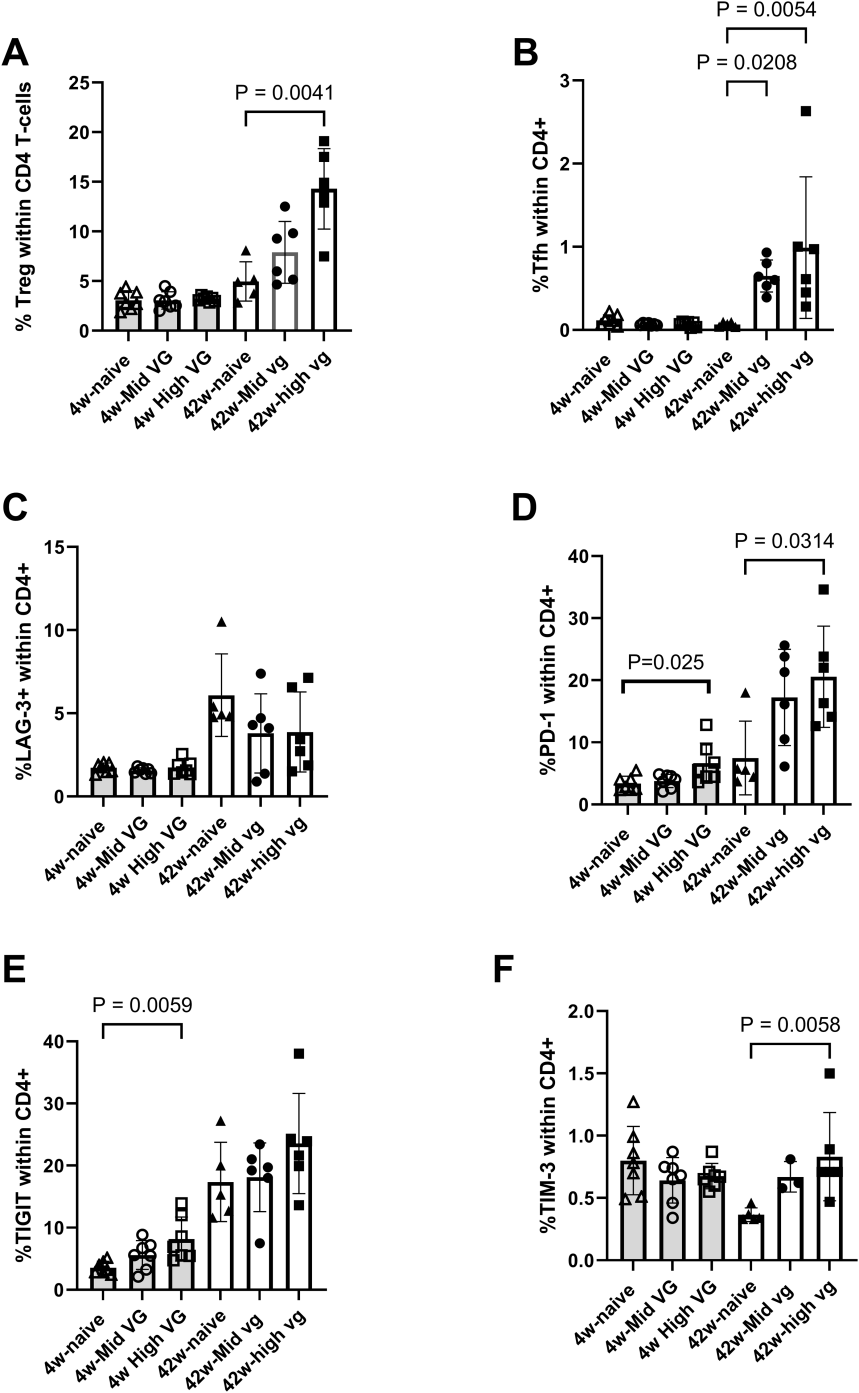
Flow cytometry demonstrates expression of exhaustion markers on liver CD4 T cells isolated from female AAV-HBV-transduced mice. **(A)** Percentage of regulatory T cells and follicular helper T cells within the CD4 T-cell population. **(B)** Percentage LAG-3+, **(C)** PD-1+, **(D)** TIGIT+, and **(E)** TIM-3+ CD4 T cells. Data are shown as mean with errors (SD) from a single experiment run. Kruskal–Wallis test was performed across the three groups of each study, and significant comparisons are highlighted. AAV-HBV, adeno-associated virus–hepatitis B virus.

In the CD4 T-cell compartment, there was an increased frequency of both regulatory T cells (Treg) and T-follicular helper (Tfh) cells in the mice that were transduced with high titer of AAV-HBV for 42 weeks compared to naïve mice (p = 0041 and p = 0.0054, respectively) and compared to mice transduced with AAV-HBV for 4 weeks (**Figures 5A, B**). CD4 T cells did express exhaustion markers. In mice transduced with AAV-HBV for 4 weeks, the frequency of TIGIT+ and PD-1+ CD4 T cells was significantly greater in the high-titer mice (p = 0.0059 and p = 0.025, respectively, **Figures 5E, D**). In mice that were transduced with AAV-HBV for 42 weeks, the frequency of PD-1+ and TIM-3+ CD4 T cells was significantly greater in high-titer mice compared to naïve mice (p = 0.0314 and p = 0.0058, respectively, **Figures 5D, F**). The frequency of LAG-3+ and TIGIT+ CD4 T cells was not increased (**Figures 5C, E**). For CD4+ Treg and Tfh cells, there was a discrepancy between RNA-sequence analysis and immunophenotyping results. This was likely due to the low frequency of these cells and the low number of cells used for single-cell RNA sequencing compared to flow cytometry. Overall, the immunophenotyping data confirm our scRNA-sequencing data that more exhausted T cells are detectable in the liver of mice transduced with AAV-HBV.

### Human atlas and translatability of AAV-HBV model CD8 T-cell exhaustion with IT and IA CHB stages of disease

Using the cell typing approach described above for the scRNA-sequencing analyses of the AAV-HBV-transduced mice, a human liver atlas was built from previously published single-cell data, and similar analyses identified a CD8 T-cell exhausted population in human liver samples from IT and IA CHB donors (30). The human Tex expressed *PDCD1*, *TOX*, *TIGIT*, *GZMK*, and *LAG3* and no *TCF7*, similar to the mouse Tex (**Supplementary Figure S10**). Additionally, the human Tex also expressed *LAYN* and *CTLA4*, which were not detected in mouse cells (**Supplementary Figure S10**).

**Figure 6:**
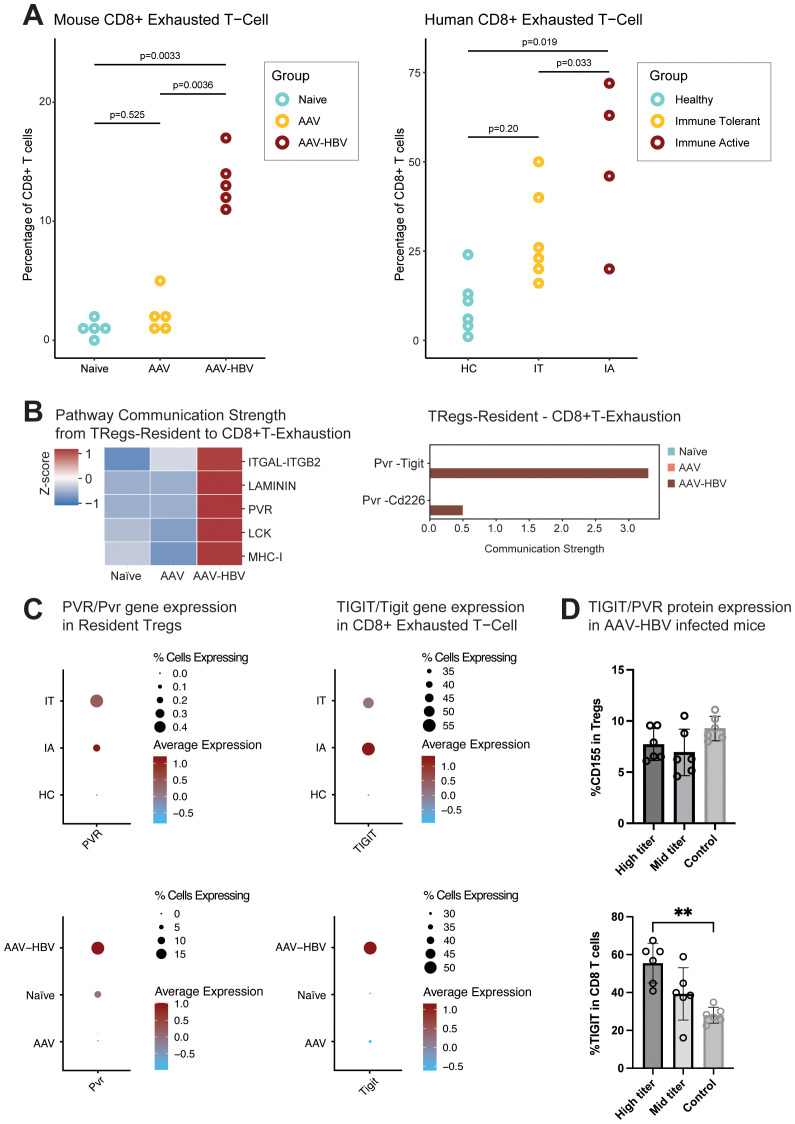
CD8 exhaustion is present at mRNA and protein levels in the liver of AAV-HBV-transduced mice and at mRNA levels in human HBV liver samples. **(A)** Proportion of the CD8 exhausted T cells across the mouse and human sample groups. The human samples are divided into healthy controls (HC), immune-tolerant (IT) donors, and immune-active (IA) donors. Statistical comparisons were conducted using a non-parametric Wilcoxon test and Bonferroni correction. **(B)** Heatmap from the cell–cell communication strength z-score for pathway communication between resident Tregs and exhausted CD8 T cells. Barplot for the *Pvr* pathway interaction strength between the different mouse groups. **(C)** Dotplot of scaled data of the gene expression of *Pvr*/*PVR* and *Tigit*/*TIGIT* across the different mouse and human sample groups. **(D)** Barplot with the frequency of protein expression from flow cytometry analysis. Percentage of CD155 (PVR) in the Tregs and percentage of TIGIT protein expression in CD8 T cells from AAV-HBV-infected mice. Data are shown as mean with errors (SD) from a single experiment run with group sizes of 7. Statistical comparisons were conducted using a non-parametric Mann–Whitney test. Comparisons with p-value <0.05 are shown with (*). AAV-HBV, adeno-associated virus–hepatitis B virus.

After the identification of cell types, using an iterative subclustering approach, the frequencies of Tex in the human liver across the different HBV disease stages were assessed and compared. The IA donors showed an increased frequency of Tex compared to IT (Wilcoxon rank-sum test, p = 0.033) donors and healthy control donors (HC) (Wilcoxon rank-sum test, p = 0.019). An increased frequency of Tex was consistent with the observation in the CD8 T-cell population in the AAV-HBV model after a prolonged (24-week) transduction time (**Figure 6A**).

To understand the potential of cell–cell interactions between Tex and other cell types, we used CellChat, which makes use of a pre-defined database of receptors and ligands. The algorithm predicts significant communications by identifying differentially overexpressed ligand and receptor pairs for each cell population (37). Deconvolution of cell–cell signaling pathways showed that resident Tregs showed strong communication strength with the CD8 Tex (**Figure 6B**). After the identification of this cell–cell interaction, the pathways driving the cell–cell communication were explored (**Figure 6B**), identifying the poliovirus receptor (PVR) as an upregulated pathway. This pathway encompassed both Pvr–Tigit and Pvr–Cd226 receptor–ligands (**Figure 6B**). Interestingly, the Pvr–Tigit interaction between resident Tregs and CD8 Tex showed an increased communication score in AAV-HBV, but not in AAV control or naïve mice (**Figure 6B**).

The follow-up study in AAV-HBV mice through flow cytometry showed that PVR was detected in Tregs from mice infected with high (7.7% ± 1.6 SD) and mid titer (6.9% ± 2.3 SD) and controls (9.3% ± 1.2 SD) (**Figure 6D**). No significant differences were observed across the three groups. However, within the CD8 compartment, a significant increase in the proportion of CD8s expressing TIGIT was observed in the high-titer mice compared to the control naïve mice (**Figure 6D**).

In CD8 Tex from the AAV-HBV arm, an increased average expression of *Tigit* together with increased *Pvr* expression in resident Tregs (**Figure 6C**, **Supplementary Figure S11**) was observed. Across the human samples, *TIGIT* was only expressed in the IT and IA samples, but not in healthy controls or chronic resolvers (**Figure 6C**). Similarly, in analyses of Tregs, *PVR* expression was only seen in IT and IA samples.

## Discussion

In this study, a mouse and human liver atlas of intrahepatic T cells was constructed using single-cell RNA-sequencing data from a long (24-week) AAV-HBV transduction model as well as published data from human samples from CHB patients at IT and IA stages of infection. The single-cell observations were generally confirmed through flow cytometry on AAV-HBV mouse IHICs, and the findings were also consistent with previously published human data. Our initial approach (with the 24-week study) to isolate IHICs from the liver using mechanical digestion resulted in good recovery of the T-cell and B-cell compartments but negatively impacted the recovery of a fragile CD45− fraction of cells, such as Kupffer cells and LSECs. Their recovery was improved using enzymatic digestion, which we performed in the follow-up 4- and 42-week studies (38). Although not significantly different, the uneven distribution of neutrophils across study groups may be due to technical capture issues of the technology (**Figure 1D**, **Supplementary Table S4**) as previously described (39).

Chronic viral infections lead to T-cell exhaustion, and the LCMV mouse model has been extensively used to investigate this link. The more recently established and expanded use of the AAV-HBV mouse model may benefit from in-depth characterization of the immune liver environment and, in particular, the T-cell exhaustion marker levels and phenotype in the AAV-HBV mouse model. Studies in the LCMV mouse model have identified heterogeneity in Tex phenotypes, which are characterized by distinct surface receptors, functionality, proliferative capacity, and tissue localization during chronic viral infections (13, 39–41). Interestingly, the pool of Tex has been shown to be replenished by precursor-like T cells (Tpex), which exhibit self-renewal capacity and are TCF-1 dependent. Tpex are characterized as PD-1+, TOX+, and TCF-1+ (TCF-7 gene), whereas Tex are characterized as PD-1+, LAG-3+, TIGIT+, TOX+, and TCF-1− (40). Similar to the LCMV model, we found both Tpex and Tex in liver samples from 24-week AAV-HBV-transduced mice but only Tex in mice transduced for 4 weeks, although the Tpex pool was similar in the AAV-HBV, AAV control arms, and naïve mice. Tpex have been described as critical in maintaining the pool of exhausted T cells (42). The transcription factor TOX may be critical in identifying this Tpex population since the absence of TOX has been described to lead to a reduction in the Tpex population (43–45). Notably, in this study, an increase in TOX-expressing CD8 T cells was observed in mice transduced for 42 weeks with a higher AAV-HBV titer, suggesting that these may be distinct from Tpex and contribute to the establishment and maintenance of the exhaustion phenotype observed in the (high-titer) AAV-HBV model. Moreover, in the absence of antigen, it is postulated that Tpex transition into conventional memory-like cells with self-renewing capacity (40, 42). Importantly, we used *Tcf7* (encoding TCF-1) to distinguish between terminally exhausted T cells and the pre-exhaustion phenotype (**Figure 2C**). TCF-1 has been linked to cell renewal capacity and effector function in chronic infection, which is lost upon transition to a fully exhaustion phenotype (35, 42, 46, 47). To determine the signature of exhaustion, phenotypic flow cytometry frequency profiles of IHICs from control and AAV-HBV mice were compared. These data showed that among total CD8 T cells, Tex, but not Tpex, could be identified in higher frequency in AAV-HBV-infected mice compared to control mice, suggesting that by 24 weeks post-transduction, CD8 T cells have become phenotypically exhausted in the AAV mouse model. A limitation of these AAV-HBV mouse studies assessing overall T-cell populations is that they do not provide any insight into HBV antigen-specific T cells. Even though studies have described HBV-specific T cells in humans (23) and mice (48), the limited availability of reagents covering multiple HBV proteins makes it challenging to study this antigen-specific population. We therefore cannot exclude the possibility that the observed exhaustion marker(s) may have origin(s) from non-HBV-specific T cells (49). More recent findings have also highlighted the diversity of HBV-specific T-cell dysfunction (24), highlighting the complexity of the disease and potential translatability to preclinical models.

The exhaustion phenotype is a continuous phenotype (50, 51), and it is challenging to categorize different states of exhaustion with flow cytometry, as we are limited to a smaller set of protein markers. This is a limitation of the technology, even though it allows for a simple cell type delineation based on protein marker association. The follow-up flow cytometry assessment of samples from the high-titer 42-week AAV-HBV arm showed the presence of Tex although at a lower frequency than observed with single-cell RNA sequencing (it is well established that mRNA expression only moderately correlates with protein expression) (52). An unrelated study has shown that in CD8 T cells, the differences between mRNA and protein are independent of T-cell differentiation or activation status (52). Moreover, the assignment of Tex in flow cytometry gating is conducted on a cell-by-cell level, whereas single-cell RNA sequencing is conducted based on clustering approaches and could inflate frequencies.

In comparing mice that were transduced with AAV-HBV for 4 or 42 weeks, the longer transduction had a higher frequency of exhausted T cells. Apparently, a higher titer and a longer AAV-HBV transduction induce more pronounced exhaustion marker expression of PD-1, TIGIT, LAG3, and TCF-1. The only marker that was significantly changed was LAG-3. This observation aligns with previous studies indicating that T-cell exhaustion is not solely dictated by the expression levels of individual inhibitory receptors but rather by the cumulative impact of multiple co-inhibitory molecules and transcriptional changes (53, 54). While PD-1, TIGIT, and Tim-3 are hallmark markers of exhaustion, their expression can reach a plateau in chronic infections and persistent antigen exposure, as seen in HBV models (20, 55). LAG-3, however, has been identified as a key player in reinforcing exhaustion and sustaining dysfunction in chronic viral infections (56). It is particularly upregulated in settings of prolonged antigen exposure and is associated with deeper exhaustion states, where T cells exhibit greater dysfunction and reduced responsiveness to reinvigoration strategies (57). In chronic HBV infections, LAG-3 is increasingly recognized as an important regulator of CD8^+^ T-cell dysfunction, possibly compensating for the roles of PD-1 and TIGIT in sustaining Tex phenotypes (58). Thus, our findings suggest that LAG-3 may serve as a primary exhaustion-associated molecule in this model, potentially acting as a dominant inhibitory receptor over PD-1, TIGIT, or TIM-3 in the context of AAV-HBV-induced chronic liver infection. Moreover, since the exhaustion phenotype could be observed both in single-cell RNA sequencing at 24 weeks and in flow cytometry at 42 weeks, it suggests that time post-transduction and perhaps titer (i.e., HBV replication) are key parameters. In a study looking at HBV-specific CD8 T cells from HBV donors, TOX levels were higher in CD8+ T cells from chronic HBV donors versus resolved patients (59). Moreover, in the LCMV mouse model, TOX expression is dependent on the antigen dose, which promotes T-cell dysfunction and long-term survival (44). Finally, our data showed a significantly higher proportion of CD4 Tregs in high-titer mice transduced for 42 weeks compared to mice transduced for 4 weeks (**Figure 4A**), which is consistent with human data wherein a higher frequency of Tregs was observed in chronic HBV-infected individuals relative to healthy controls (60). Furthermore, inhibitory receptor molecule expression on liver-resident CD4 T cells from AAV-HBV-transduced mice is comparable to that described in the literature relating to CHB patients (61).

To the best of our knowledge, this is the first study using single-cell RNA sequencing on IHICs from AAV-HBV-infected liver samples and its comparative assessment of scRNA sequencing of human CHB samples. A previous study in a different mouse model (using Alb-Cre transgenic mice transduced with AAV-rcccDNA) demonstrated the presence of exhaustion after 8 weeks (62). This exhausted population also expressed *Pdcd1* and *Tox*, but only reached a frequency of 1.49% of total CD8 T cells (62). The difference between that published study and our data may be due to either the shorter period of transduction or a milder exhaustion phenotype in the Alb-Cre transgenic mouse model.

Various studies have shown that peripheral T cells (16, 63) or HBV-specific T cells from HBV-infected individuals are exhausted (14, 16, 63). A recent study has further investigated the immune landscape in the liver of CHB patients from different stages of disease at the single-cell level (30). These liver biopsy data were re-analyzed, and the same exhaustion markers were identified from our current study in total CD8 T cells from AAV-HBV mice as previously observed in samples from IT and IA HBV-infected individuals, suggesting that the immunological phenotype of the AAV-HBV mouse model shares features of the human disease. In both cases, total T cells in the liver were examined, which are likely similar to T cells recruited to the liver by various inflammatory stimuli, with the possibility of the presence of some HBV-specific T cells. Moreover, the T-cell dysfunctions observed in our model are likely due to classical exhaustion mechanisms driven by persistent antigen stimulation and do not fully recapitulate the multifaceted impairments seen in CHB subjects, especially those stemming from suboptimal T-cell priming in the absence of innate immune activation.

More recently, tools to further understand intercellular communications from single-cell RNA-sequencing data have been developed (37, 64). Zhang et al. described a potential interaction between Tregs and Tex and the increased frequency of both populations in IT and IA donors, and we aimed to understand the same in the AAV-HBV model (30). The analytical cell–cell communication tool CellChat encompasses a manually curated database of ligand–receptor interactions, which were corralled into pathways. An interesting correlation between Tregs–Tex and the PVR–TIGIT axis was observed. It is well established that *TIGIT* is expressed by T cells, such as exhausted T cells and Tregs. *PVR* was thought to be expressed by antigen-presenting cells and tumor cells (65). In the analyzed data, even though *Pvr*/*PVR* was expressed in a small percentage of the Tregs from both AAV-HBV mice and IT and IA human samples, it was consistently observed across the two studies (**Figure 6C**). We used flow cytometry in AAV-HBV mice to further validate this finding, and we observed that a comparable percentage (7%–8% PVR expression in Tregs) of protein expression was observed (65). The fact that a small percentage of Tregs express PVR may have implications for cell–cell interaction and warrants further investigation. PVR binds TIGIT and, as a consequence, induces an immunosuppressive and non-cytotoxic profile (65). It remains to be seen whether these Tregs can further enhance the immunosuppressive phenotype of Tex. Finally, Tregs were significantly increased in frequency in high-titer mice vs. controls in the flow cytometry assessment but not in the single-cell analysis (possibly due to low cell numbers in single-cell analysis), which was also described in IT and IA donors (30). Importantly, the levels of antigenemia (i.e., HBsAg levels) in the high-titer AAV-HBV mouse model are more representative of the levels observed in CHB patients, providing confidence about the translatability of the described liver immune environment.

The persistence of HBV as measured by HBsAg and HBeAg apparently upregulate exhaustion markers LAG-3, suggesting a higher exhaustion burden and less functional T cells. In chronic HBV patients with very low HBsAg levels (<100 IU/mL), spontaneous HBsAg clearance is associated with functional and less exhausted T cells in comparison to those with high HBsAg levels (66). A limitation of the current study performed here, due to the lack of reagents, is that overall CD8 T-cell populations were analyzed rather than HBV antigen-specific T cells. Nonetheless, the consistency with observations of T-cell exhaustion markers from chronic HBV donors is notable.

These data support the use of the AAV-HBV mouse model for the study of potential therapeutic approaches toward the reversal of T-cell exhaustion in chronic HBV infection.

## Data Availability

The datasets presented in this study can be found in online repositories. The datasets presented in this study can be found in the GEO NCBI repository under the following accession number: GSE244487.
