# Supplementary material for: AAV-HBV mouse model replicates the intrahepatic immune landscape of chronic HBV patients at single-cell level
Source: Front Immunol. 2025 Jun 18;16:1421712. doi: 10.3389/fimmu.2025.1421712 (PMC12214900; doi:10.3389/fimmu.2025.1421712)
Supplement: Supplementary file 1 [file DataSheet1.docx]

**SUPLEMMENTARY INFORMATION**

**SUPLEMMENTARY FIGURES**

**Figure S1 gating strategy:**

**
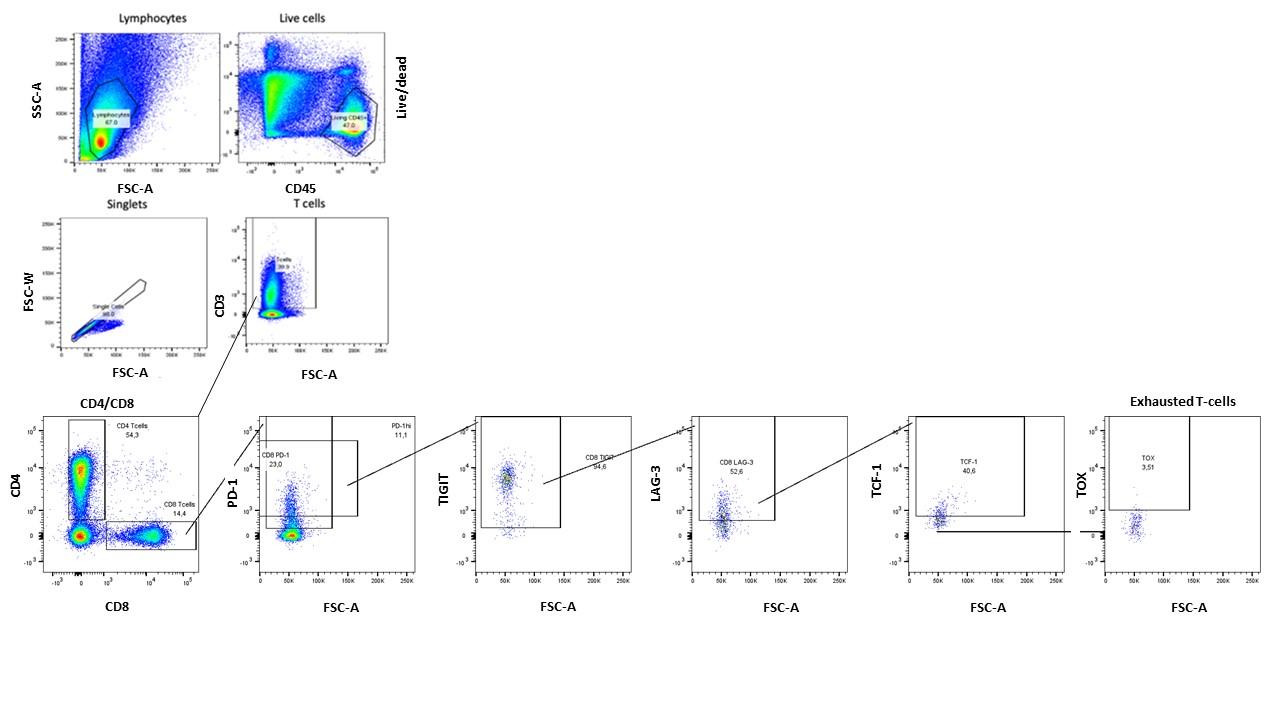
**

**Fig. S2 – Viral parameters from the different studies.**

Mean HBsAg levels over time endpoint 4 weeks post transduction (**A**), endpoint 24w post transduction (**C**), endpoint 42 weeks post transduction (**E**). Mean HBeAg levels over time endpoint 4 weeks post transduction (**B**), endpoint 24 weeks post transduction (**D**), endpoint 42 weeks post transduction (**F**). Mice transduced with AAV-HBV high titer (circles), AAV-HBV mid titer (squares) and naïve (triangles).


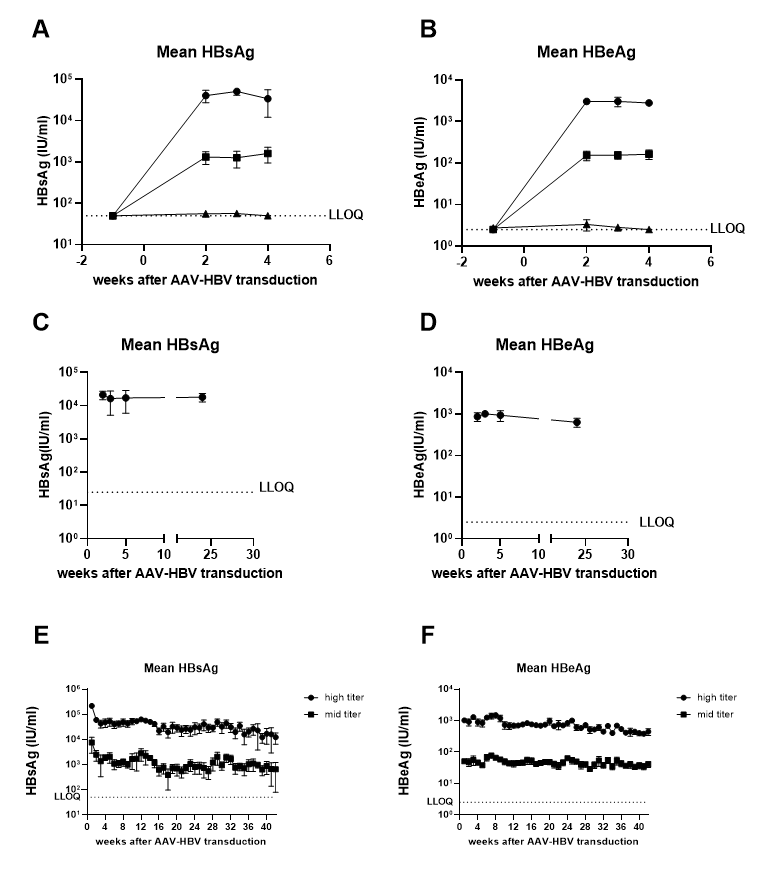


**Fig. S3 – Cell numbers of high quality (HQ) cells retrieved from each sample (single-cell RNA-sequencing)**

Cells with more than 1,000 Unique Molecular Identifiers (UMIs) or more than 200 genes or less than 25% mitochondrial counts were considered HQ cells and used for downstream analysis.

**A:** 24 week study **B:** 4-week study

**A**


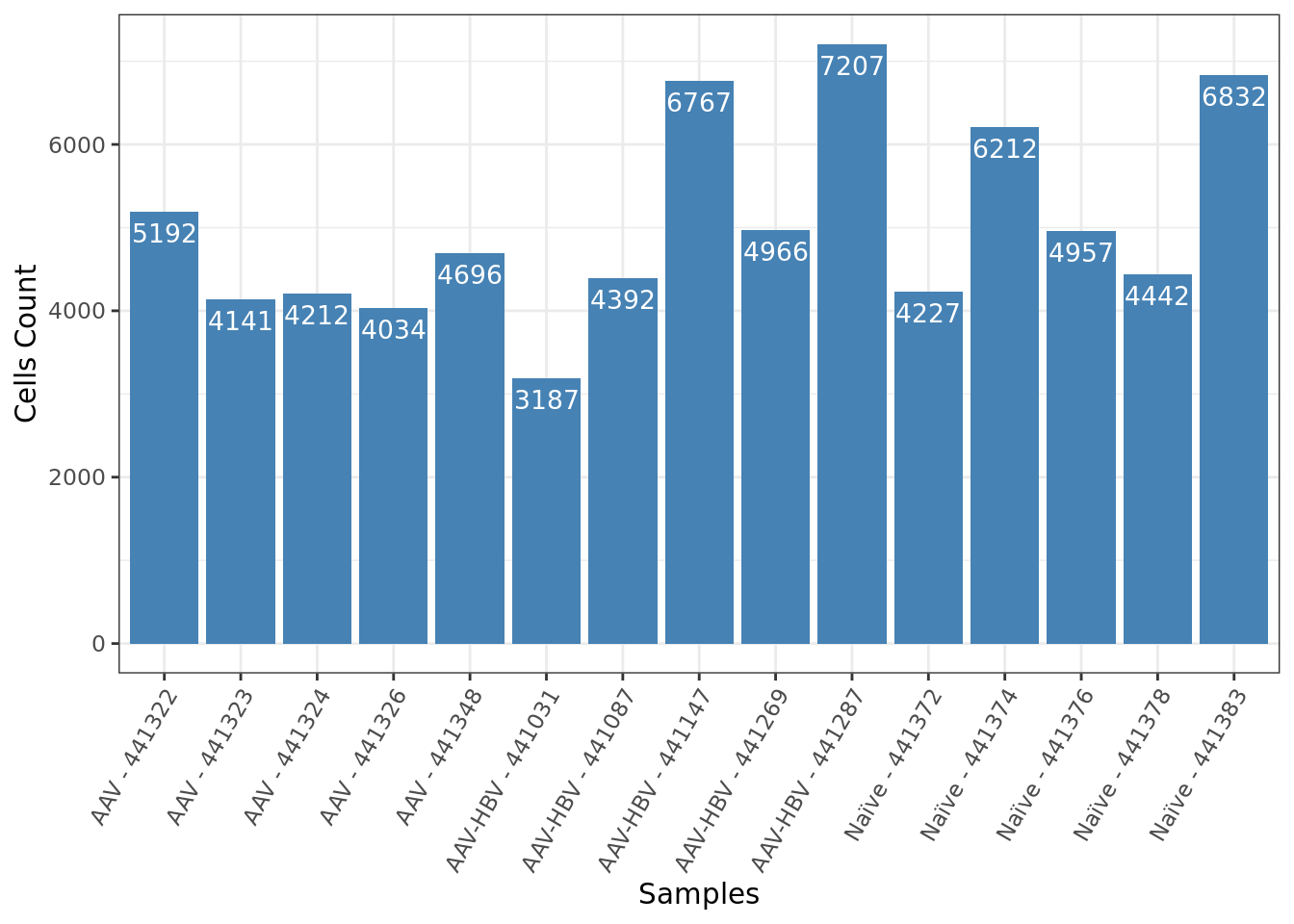


**B**


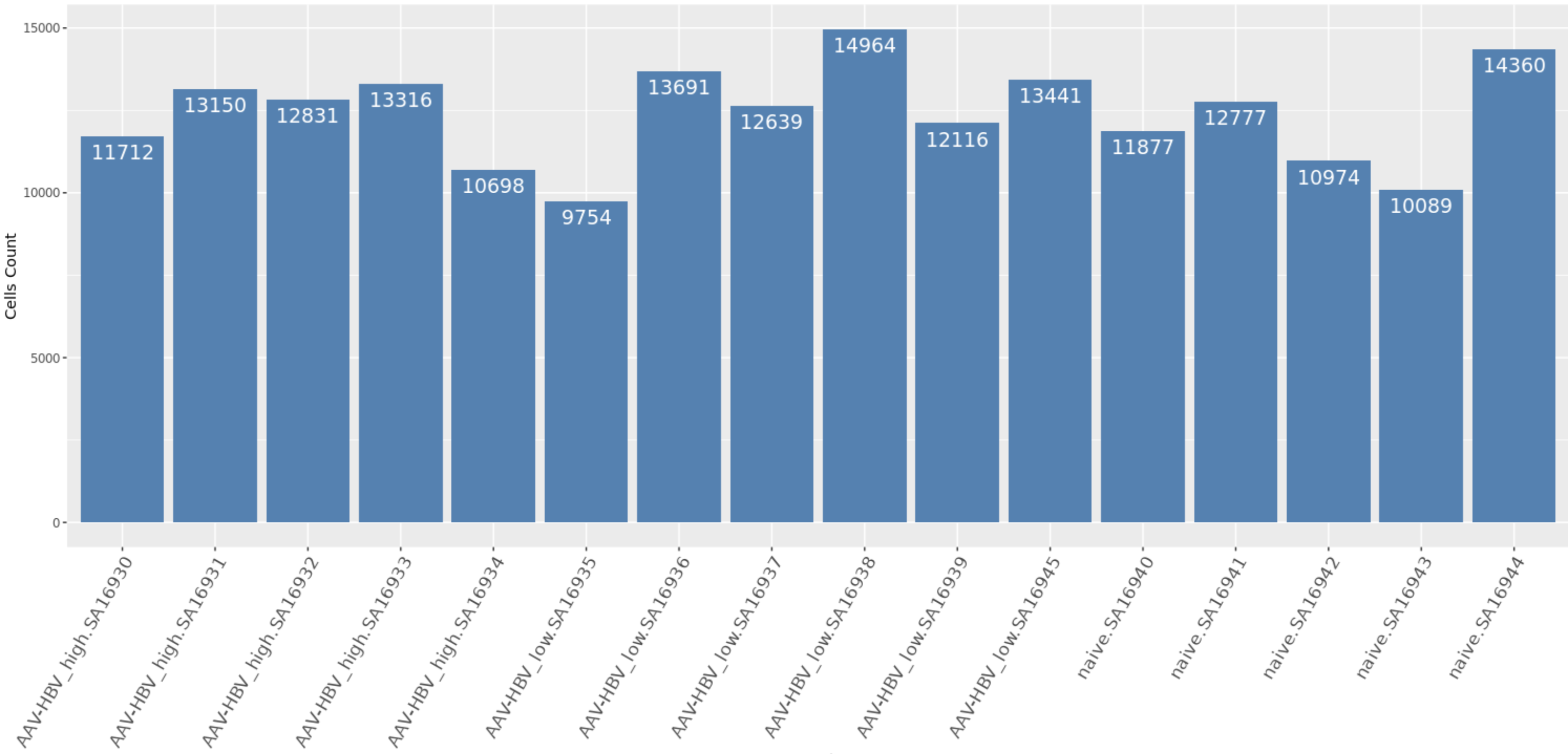


**Fig. S4 – Dendrogram with overview of cell populations**

1. across all samples (n= 15) from the 24-week single-cell study
2. across all samples (n= 18) from the 4-week single-cell study

**A**


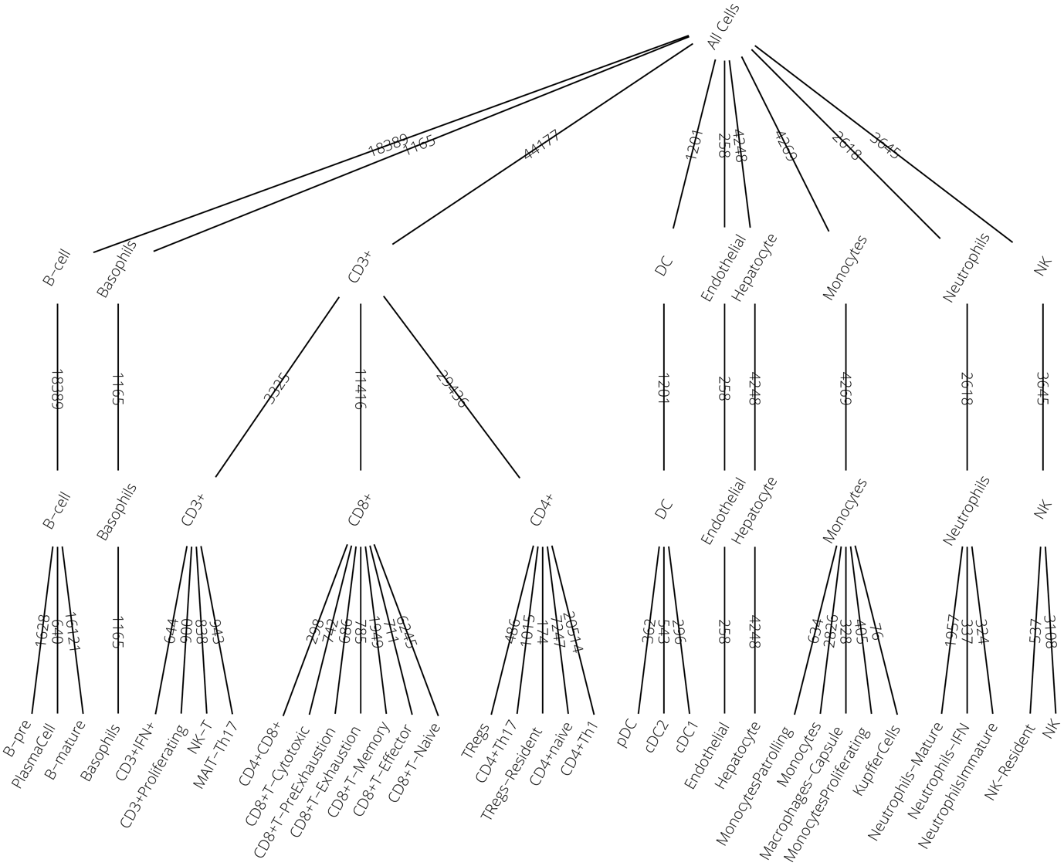


**B**

**
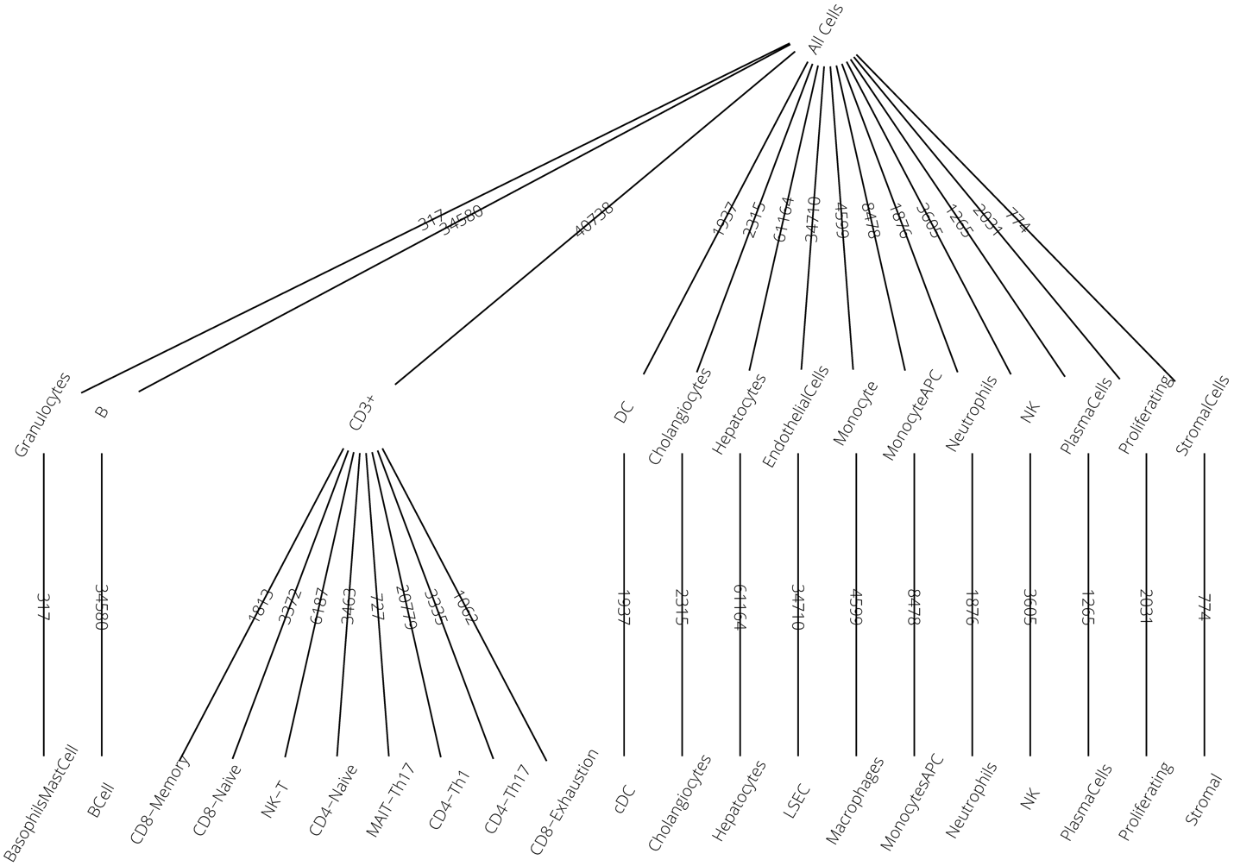
**

**Fig. S5 – Heatmap of marker genes at week 24 for**

1. **major cell populations**
2.
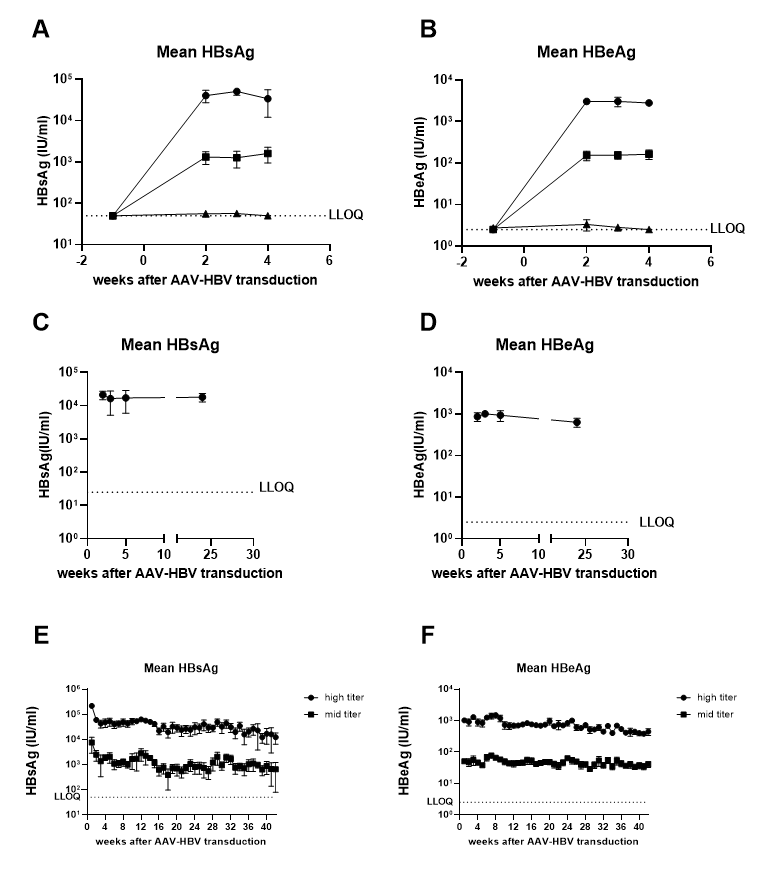

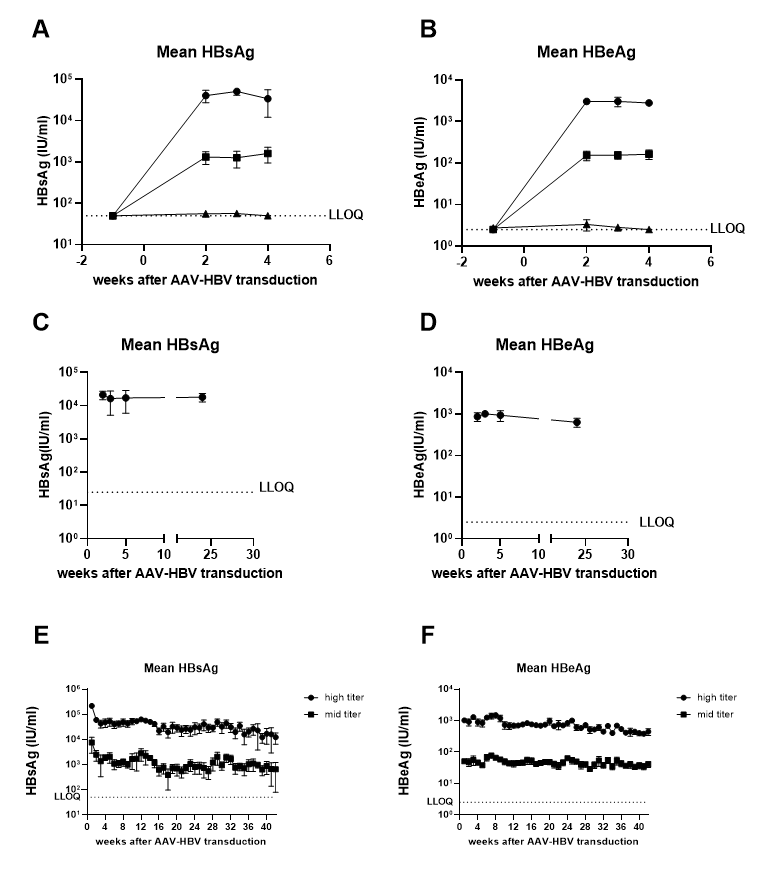

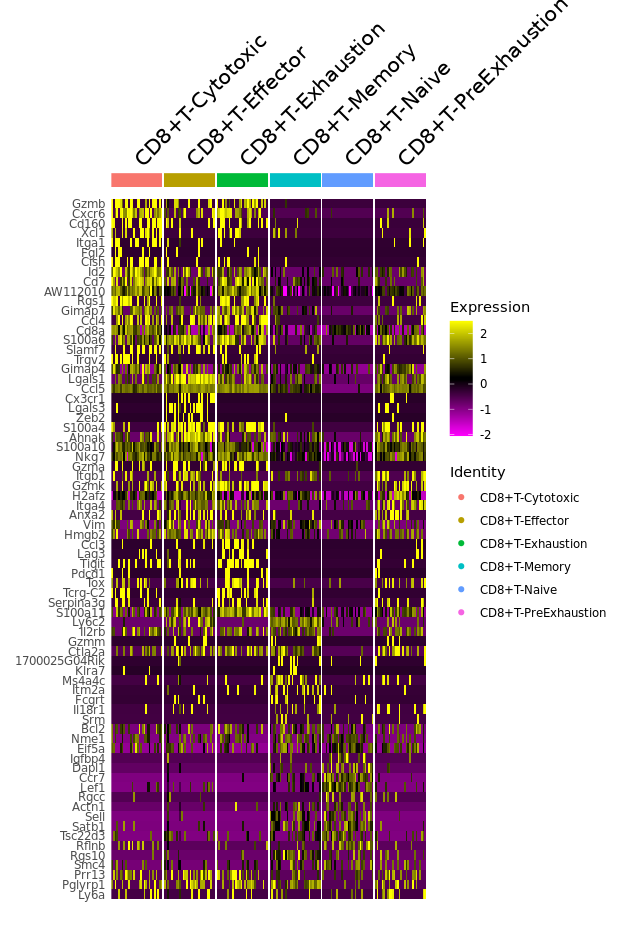

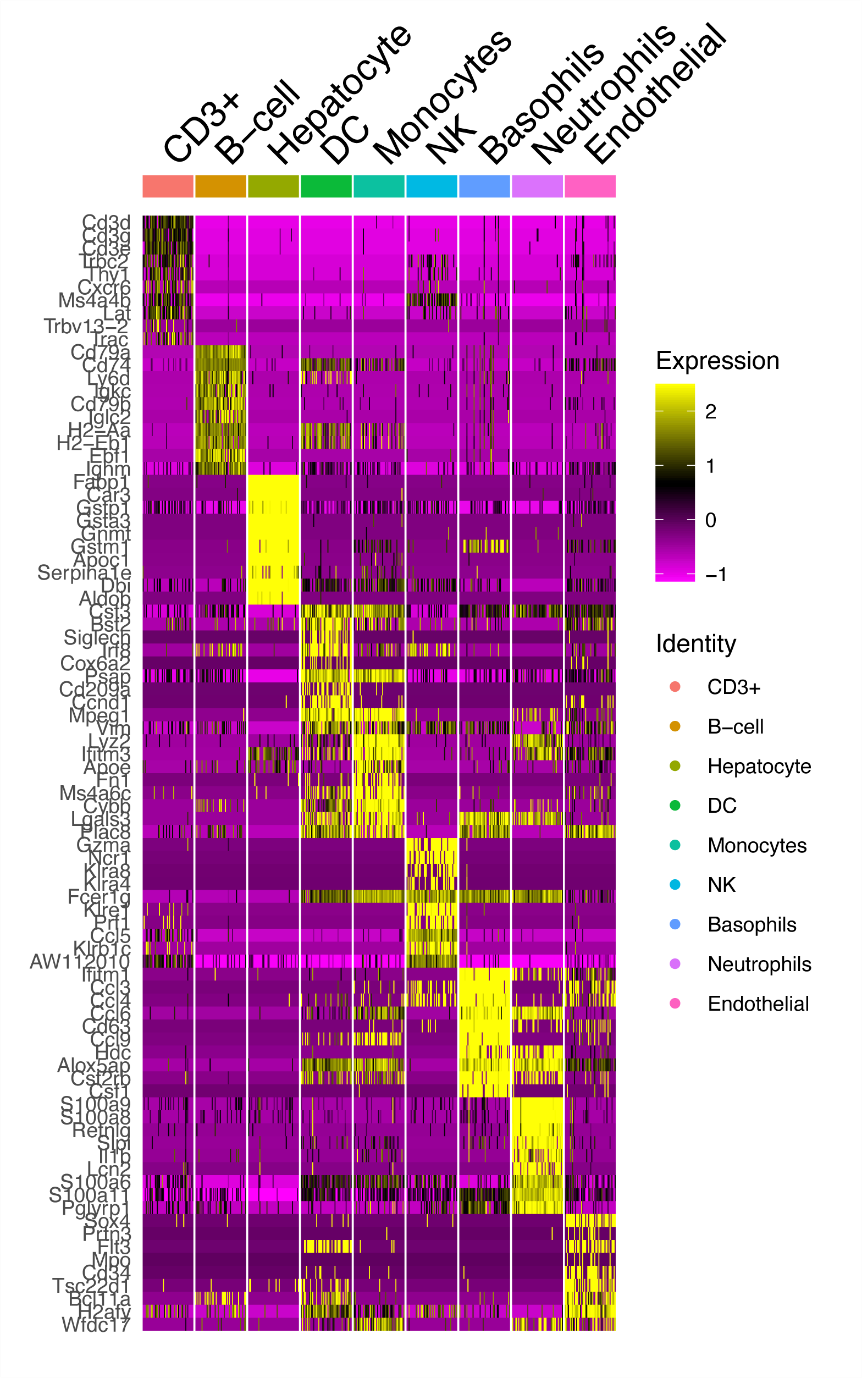
**CD8 T cell populations**

**Fig. S6 – Heatmap of marker genes at week 4 for**

1. **major cell populations**
2.
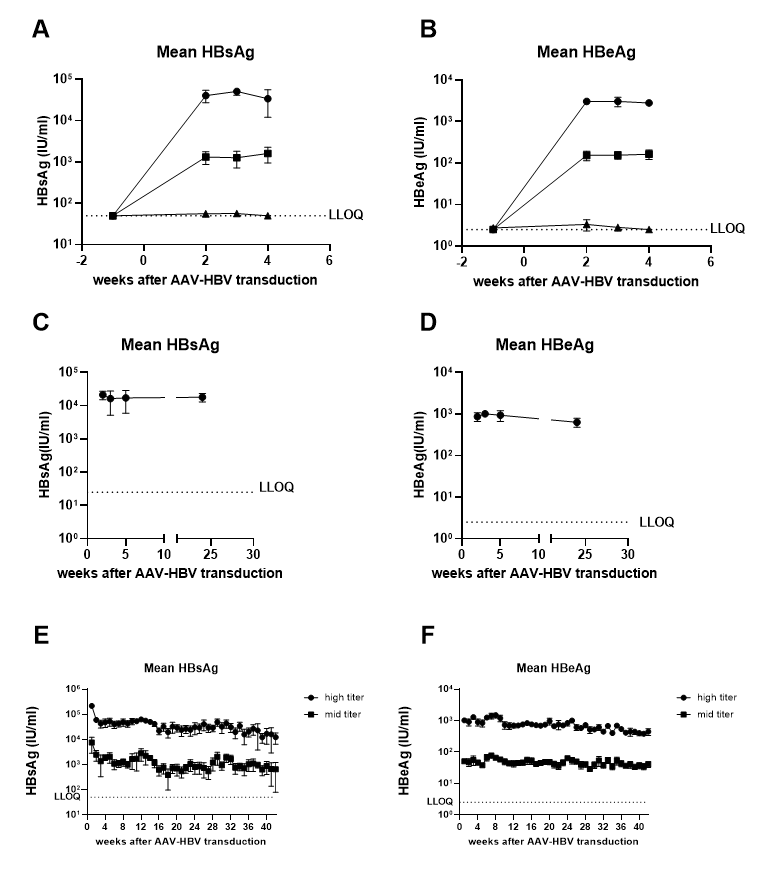

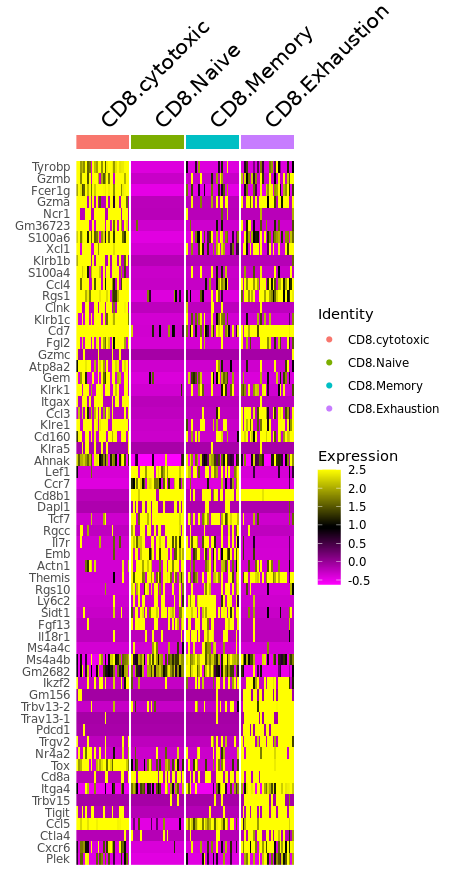

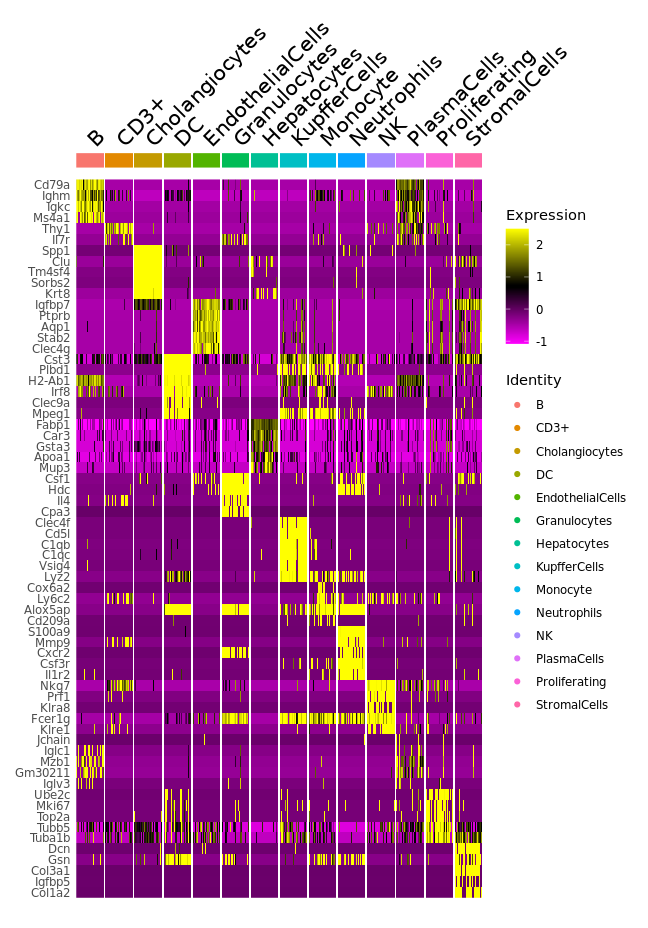
**CD8 T cell populations**


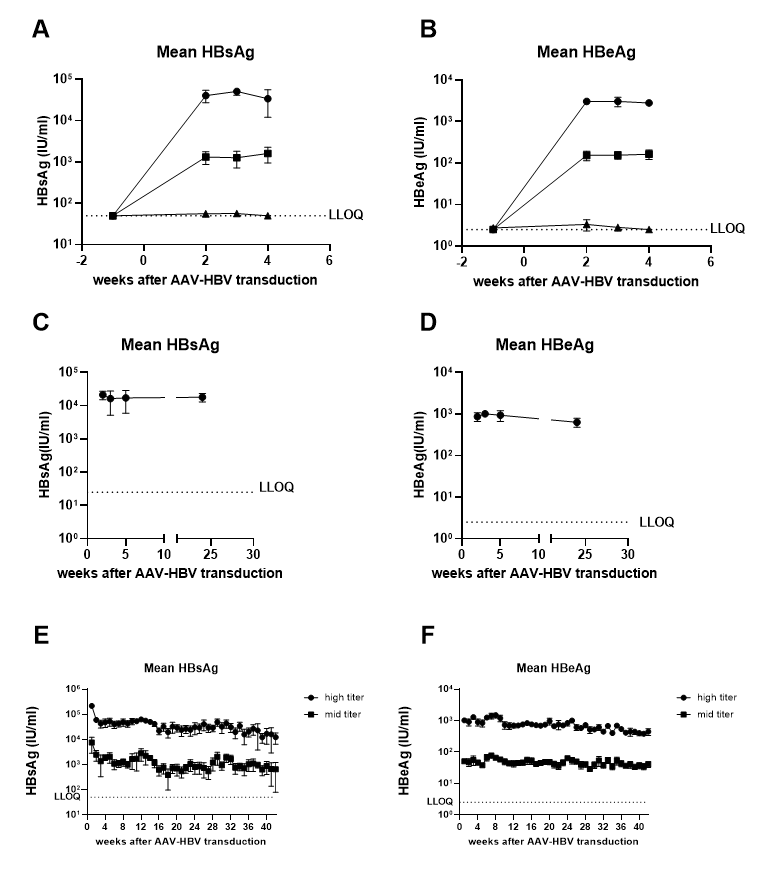


**Fig. S7 – Cell numbers per group across the difference cell types**

1. at week 24
2. at week 4

**A**


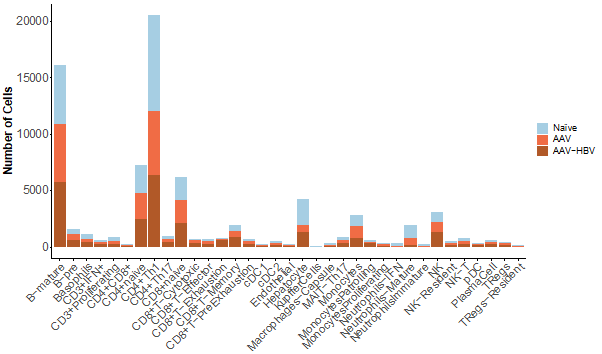


**B**


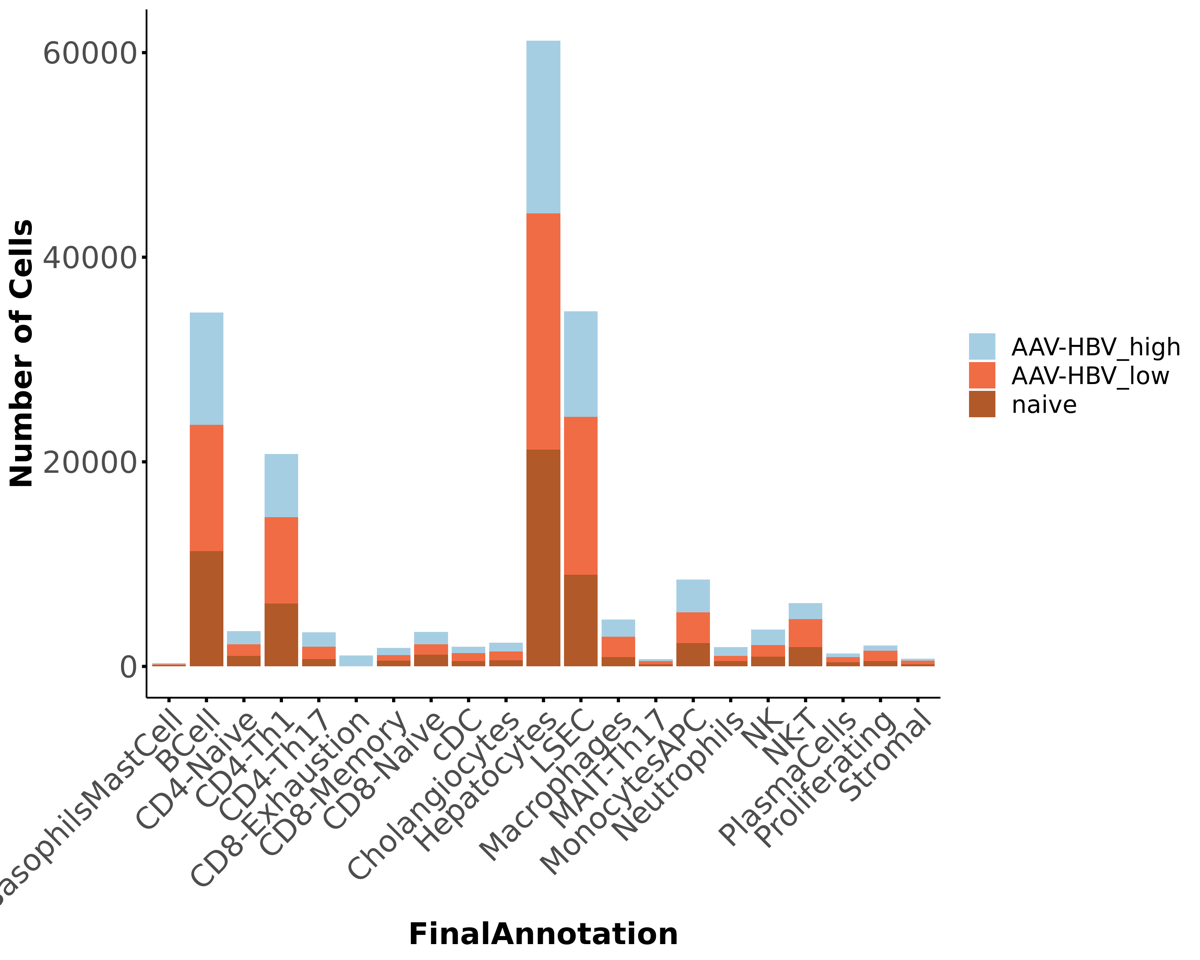


**Fig. S8 – UMAP and dotplot of CD4 T cell populations and their hallmark genes at week 24**


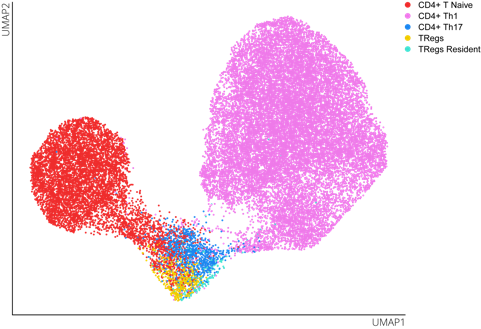

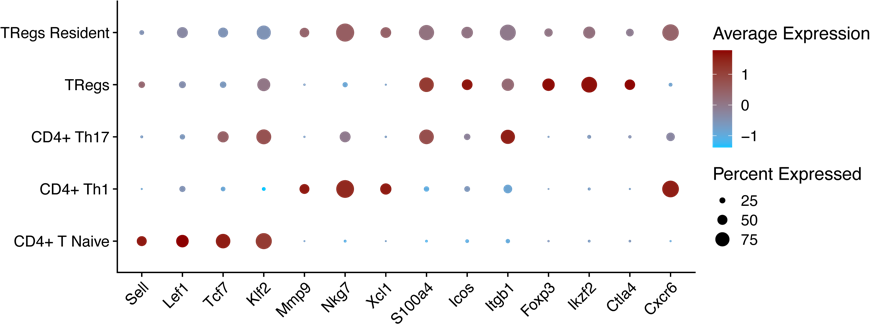


**Fig. S9 – Frequency of the CD8 Pre-exhausted T cells across the different mouse groups (week-24)**


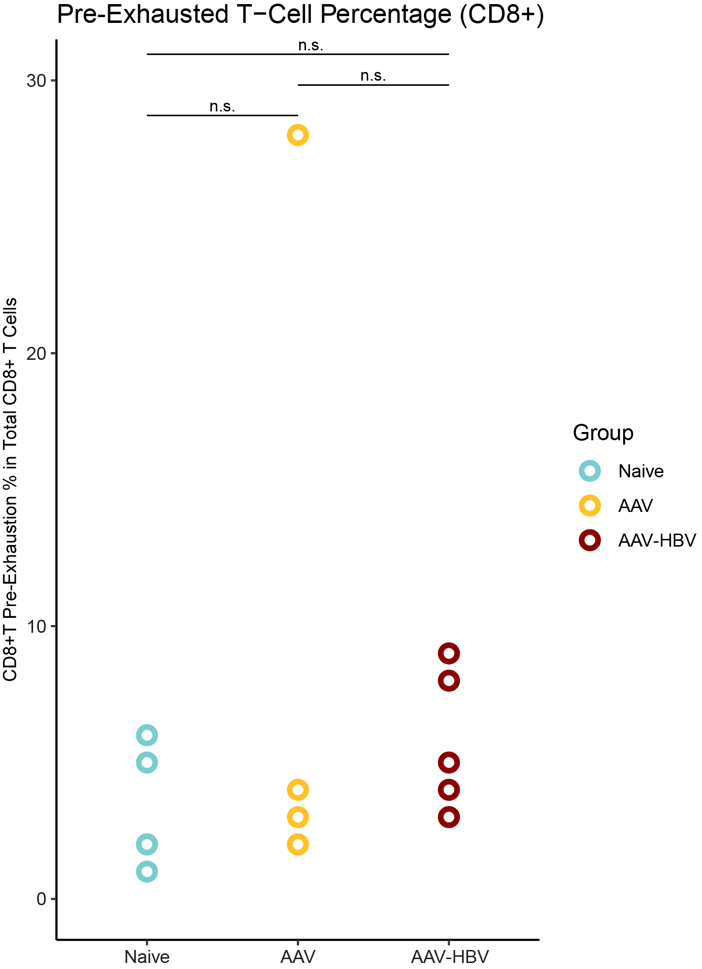


**Fig. S10 – Exhaustion marker expression across the different CD8 T cell subpopulations**


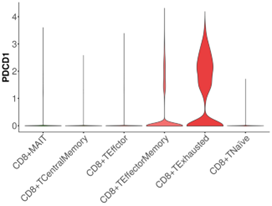

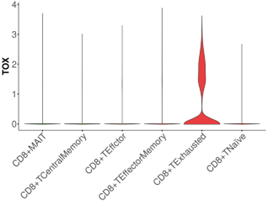


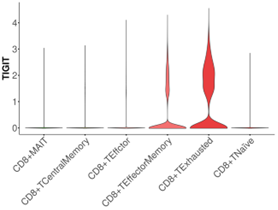

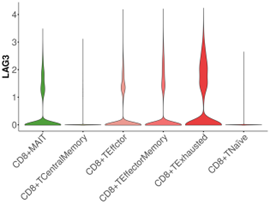


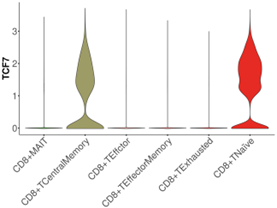

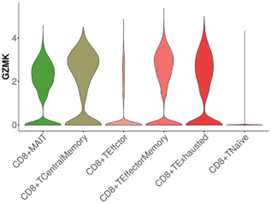


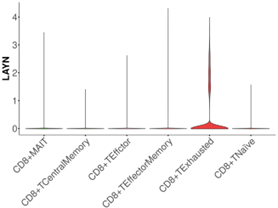

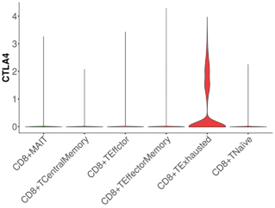


**Fig. S11 – Pvr expression across cell types in AAV-HBV**


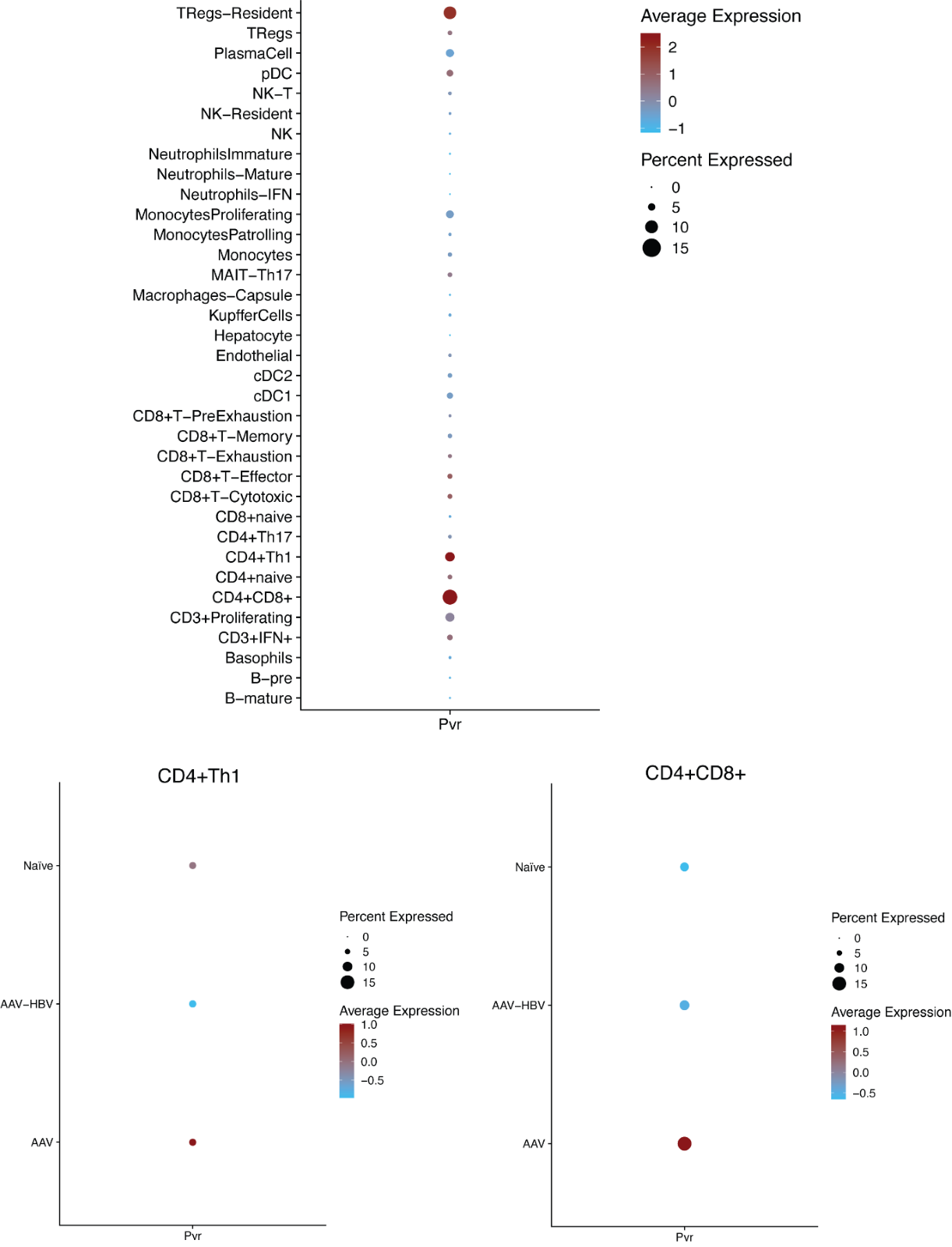


**SUPPLEMENTARY TABLES**

**Supplementary table 1 – Marker genes for cell population typing**

| ***Cell Type*** | ***Marker genes*** |
| --- | --- |
| CD8 naïve | *Sell, Ccr7, Lef1, Tcf7, Klf2* |
| CD8 effector | *Gzma, Gzmb, Ccl5, Cx3cr1, Klrg1, S1pr5* |
| CD8 memory | *Sell, Ccl5, Ccr7, Eomes, Tcf7, Ly6c2* |
| CD8 cytotoxic | *Gzmb, Ccl5, Ccl4, Il7r, Gzma, Ccl3, Cd160* |
| CD8 precursor exhaustion | *Gzmk, Eomes, S100a6, Tox, Tigit, Klf2, Tcf7* |
| CD8 terminal exhaustion | *Gzmk, Lag3, Tox, Tigit, Pdcd1, Eomes, Nr4a2, Il10ra* |
| CD4 naïve | *Ccr7, Tcf7, Lef1, Sell, S1pr1, Ly6c1* |
| TRegs | *Foxp3, Ikzf2, Ctla4, Tigit, Capg, Tnfrsf4* |
| Tregs resident | *Cxcr6, Foxp3, Ikzf2, Ctla4, Tigit, Capg, Tnfrsf4* |
| Th1 | *Cxcr6, Ly6a, Il2rb, Klrb1c, Mmp9* |
| Th17 | *S100a4, Icos, Vim, Capg, Itgb1* |
| Cycling T-cell | *Top2a, Tuba1b, Mki67, Stmn1* |
| IFN stimulated T-cell | *Isg15, Ifit1, Ifit3, Cxcl10, Isg20, Rsad2, Irf7* |
| MAIT Th17 | *S100a4, Il7r, Tmem176a/b, Ramp1, Capg, Rorc, Pxdc1* |
| NK-T | *Fcer1g, Ly6c2, Klra7, Trdc, Ccl5, Anxa2, Cd7* |
| pre-B | *Iglc1, Cd24a, Sox4, Rgs2, Cd93* |
| Mature B | *Cd19, Cd79a, Cd79b, Ms4a1, Ly6d* |
| Plasma Cell | *Jchain, Igha, Igkc, Iglc1* |
| cDC1 | *Clec9a, Cd24a, Wdfy4, Id2, Ppt1* |
| cDC2 | *Cd209a, Cd7, Klrd1, Lgals1* |
| pDC | *Bst2m Siglech, Ly6d, Cox6a2* |
| Endothelial | *Prtn3, H2afy, Sox4* |
| Hepatocyte | *Fabp1, Car3, Apoe, Alb* |
| Monocytes | *Lyz2, Chil3, Hp, F13a1, Gn1* |
| Macrophages Capsule | *C1qa, C1qb, C1qc, Cd81, Apoe* |
| Monocytes Patrolling | *Ace, Ear2, Eno3, Gngt2* |
| Kupffer Cells | *C1qa, Vsig4, Cd5l, Folr2, Timd4, Clec4f* |
| Neutrophils Mature | *Cxcr2, Fcgr3, Csf3r, S100a8* |
| Neutrophils IFN | *Cxcr2, Rsad2, Isg15, Oasl1, Ifit1, S100a8* |
| Neutrophils Immature | *Ltf, Padi4, Lcn2, Camp, Mmp8, Ly6g, S100a8* |
| Basophils | *Cd63, Gata2, Csf1, Fcer1a, Cpa3, Hdc* |

**Supplementary Table 2 – Cell Population Numbers and Frequencies identified in 24 week study**

| ***Cell Population*** | ***Cell Numbers*** | ***Total Frequency across all groups (%)*** | ***AAV-HBV*** | ***AAV-control*** | ***Naïve*** |
| --- | --- | --- | --- | --- | --- |
| **B-mature** | 16121 | 20 | 5761 | 5121 | 5239 |
| **B-pre** | 1628 | 2 | 646 | 510 | 472 |
| **Basophils** | 1165 | 1.5 | 418 | 294 | 453 |
| **CD3+IFN+** | 644 | 0.8 | 320 | 169 | 155 |
| **CD3+Proliferating** | 900 | 1.1 | 310 | 205 | 385 |
| **CD4+CD8** | 298 | 0.4 | 127 | 82 | 89 |
| **CD4naive** | 7247 | 9.1 | 2508 | 2253 | 2486 |
| **CD4Th1** | 20514 | 26 | 6434 | 5584 | 8496 |
| **CD4Th17** | 1015 | 1.3 | 419 | 281 | 315 |
| **CD8T-Cytotoxic** | 742 | 0.9 | 379 | 231 | 132 |
| **CD8T-Effector** | 711 | 0.9 | 317 | 223 | 171 |
| **CD8T-Exhaustion** | 785 | 1 | 646 | 99 | 40 |
| **CD8T-Memory** | 1949 | 2.4 | 863 | 558 | 528 |
| **CD8T-Naive** | 6245 | 7.8 | 2109 | 2059 | 2077 |
| **CD8T-PreExhaustion** | 686 | 0.9 | 258 | 308 | 120 |
| **cDC1** | 296 | 0.4 | 135 | 75 | 86 |
| **cDC2** | 543 | 0.7 | 211 | 146 | 186 |
| **Endothelial** | 258 | 0.3 | 99 | 69 | 90 |
| **Hepatocyte** | 4248 | 5.3 | 1317 | 686 | 2245 |
| **KupfferCells** | 76 | 0.1 | 24 | 16 | 36 |
| **Macrophages-Capsule** | 328 | 0.4 | 106 | 80 | 142 |
| **MAIT-Th17** | 943 | 1.2 | 372 | 287 | 284 |
| **Monocytes** | 2826 | 3.5 | 845 | 993 | 988 |
| **MonocytesPatrolling** | 634 | 0.8 | 334 | 159 | 141 |
| **MonocytesProliferating** | 405 | 0.5 | 130 | 115 | 160 |
| **Neutrophils-IFN** | 337 | 0.4 | 42 | 95 | 200 |
| **Neutrophils-Mature** | 1957 | 2.4 | 175 | 644 | 1138 |
| **NeutrophilsImmature** | 324 | 0.4 | 37 | 62 | 225 |
| **NK** | 3108 | 3.9 | 1350 | 858 | 900 |
| **NK-Resident** | 537 | 0.7 | 228 | 118 | 191 |
| **NK-T** | 838 | 1 | 303 | 241 | 294 |
| **pDC** | 362 | 0.5 | 176 | 67 | 119 |
| **PlasmaCell** | 640 | 0.8 | 254 | 182 | 204 |
| **TRegs** | 486 | 0.6 | 225 | 111 | 150 |
| **TRegs-Resident** | 174 | 0.2 | 57 | 49 | 68 |

**Supplementary Table3 – Cell Population Numbers and Frequencies  identified in 4 week study**

| ***Cell Population*** | ***Cell Numbers*** | ***Total Frequency across all groups (%)*** | ***AAV-HBV high*** | ***AAV-HBV low*** | ***Naïve*** |
| --- | --- | --- | --- | --- | --- |
| BasophilsMastCell | 317 | 0.16 | 90 | 102 | 125 |
| BCell | 34580 | 17.43 | 10982 | 12344 | 11254 |
| CD4.Naive | 3463 | 1.75 | 1309 | 1132 | 1022 |
| CD4.Th1 | 20779 | 10.47 | 6182 | 8460 | 6137 |
| CD4.Th17 | 3335 | 1.68 | 1400 | 1207 | 728 |
| NK-T | 6187 | 3.12 | 1576 | 2731 | 1880 |
| CD8.Exhaustion | 1062 | 0.54 | 1037 | 18 | 7 |
| CD8.Memory | 1813 | 0.91 | 696 | 569 | 548 |
| CD8.Naive | 3372 | 1.70 | 1220 | 1025 | 1127 |
| cDC | 1937 | 0.98 | 642 | 761 | 534 |
| Cholangiocytes | 2315 | 1.17 | 872 | 846 | 597 |
| Hepatocytes | 61164 | 30.83 | 16862 | 23112 | 21190 |
| LSEC | 34710 | 17.50 | 10330 | 15434 | 8946 |
| KupfferCells | 4599 | 2.32 | 1682 | 2018 | 899 |
| MAIT.Th17 | 727 | 0.37 | 220 | 303 | 204 |
| Monocytes | 8478 | 4.27 | 3186 | 3018 | 2274 |
| Neutrophils | 1876 | 0.95 | 838 | 535 | 503 |
| NK | 3605 | 1.82 | 1519 | 1130 | 956 |
| PlasmaCells | 1265 | 0.64 | 360 | 495 | 410 |
| Proliferating | 2031 | 1.02 | 496 | 1003 | 532 |
| Stromal | 774 | 0.39 | 208 | 362 | 204 |

**Supplementary table 4 –Statistics of frequencies between AAV-HBV high titer mice and AAV-control mice in all cell types in 24-week study.**

| **Cell Type** | **group1** | **group2** | **n1** | **n2** | **statistic** | **p** | **p.adj** | **p.signif** | **p.adj.signif** |
| --- | --- | --- | --- | --- | --- | --- | --- | --- | --- |
| B-cell | AAV | AAV-HBV | 5 | 5 | 11 | 0.841 | 1 | ns | ns |
| Basophils | AAV | AAV-HBV | 5 | 5 | 10 | 0.69 | 1 | ns | ns |
| CD3+ | AAV | AAV-HBV | 5 | 5 | 12 | 1 | 1 | ns | ns |
| DC | AAV | AAV-HBV | 5 | 5 | 10 | 0.69 | 1 | ns | ns |
| Endothelial | AAV | AAV-HBV | 5 | 5 | 10 | 0.69 | 1 | ns | ns |
| Hepatocyte | AAV | AAV-HBV | 5 | 5 | 12 | 1 | 1 | ns | ns |
| Monocytes | AAV | AAV-HBV | 5 | 5 | 13 | 1 | 1 | ns | ns |
| Neutrophils | AAV | AAV-HBV | 5 | 5 | 21 | 0.095 | 0.857 | ns | ns |
| NK | AAV | AAV-HBV | 5 | 5 | 5 | 0.151 | 1 | ns | ns |

**Supplementary table 5 –Statistics of frequencies between AAV-HBV high titer mice and AAV-control mice in T-cells in 24-week study.**

| **CellType** | **group1** | **group2** | **n1** | **n2** | **statistic** | **p** | **p.adj** | **p.signif** | **p.adj.signif** |
| --- | --- | --- | --- | --- | --- | --- | --- | --- | --- |
| CD3+IFN+ | AAV | AAV-HBV | 5 | 5 | 3 | 0.0556 | 0.222 | ns | ns |
| CD3+Proliferating | AAV | AAV-HBV | 5 | 5 | 4 | 0.0952 | 0.381 | ns | ns |
| MAIT-Th17 | AAV | AAV-HBV | 5 | 5 | 18 | 0.31 | 1 | ns | ns |
| NK-T | AAV | AAV-HBV | 5 | 5 | 17 | 0.421 | 1 | ns | ns |
| **CellType** | **group1** | **group2** | **n1** | **n2** | **statistic** | **p** | **p.adj** | **p.signif** | **p.adj.signif** |
| CD4+CD8+ | AAV | AAV-HBV | 5 | 5 | 10 | 0.69 | 0.69 | ns | ns |
| CD4+Th1 | AAV | AAV-HBV | 5 | 5 | 15 | 0.69 | 0.69 | ns | ns |
| Tregs Resident | AAV | AAV-HBV | 5 | 5 | 14 | 0.84 | 0.84 | ns | ns |
| CD4+naive | AAV | AAV-HBV | 5 | 5 | 14 | 0.84 | 0.84 | ns | ns |
| CD4+Th17 | AAV | AAV-HBV | 5 | 5 | 9 | 0.55 | 0.55 | ns | ns |
| TRegs | AAV | AAV-HBV | 5 | 5 | 6 | 0.22 | 0.22 | ns | ns |
| **CellType** | **group1** | **group2** | **n1** | **n2** | **statistic** | **p** | **p.adj** | **p.signif** | **p.adj.signif** |
| CD8+memory | AAV | AAV-HBV | 5 | 5 | 12 | 1 | 1 | ns | ns |
| CD8+effector | AAV | AAV-HBV | 5 | 5 | 12 | 1 | 1 | ns | ns |
| CD8+cytotoxic | AAV | AAV-HBV | 5 | 5 | 11 | 0.84 | 0.84 | ns | ns |
| CD8 precursor exhaustion | AAV | AAV-HBV | 5 | 5 | 7 | 0.31 | 0.31 | ns | ns |
| CD8 terminal exhaustion | AAV | AAV-HBV | 5 | 5 | 0 | 0.01 | 0.01 | ** | ** |
| CD8+naive | AAV | AAV-HBV | 5 | 5 | 19 | 0.22 | 0.22 | ns | ns |

**Supplementary table 6 –Statistics of frequencies between AAV-HBV high titer mice and AAV-control mice in all cell types in 4-week study.**

*AAV-HBV high vs low*

| **CellType** | **group1** | **group2** | **n1** | **n2** | **statistic** | **p** | **p.adj** | **p.signif** | **p.adj.signif** |
| --- | --- | --- | --- | --- | --- | --- | --- | --- | --- |
| B | AAV-HBV_high | AAV-HBV_low | 5 | 6 | 18 | 0.662 | 1 | ns | ns |
| CD3+ | AAV-HBV_high | AAV-HBV_low | 5 | 6 | 17 | 0.792 | 1 | ns | ns |
| Cholangiocytes | AAV-HBV_high | AAV-HBV_low | 5 | 6 | 20 | 0.429 | 1 | ns | ns |
| DC | AAV-HBV_high | AAV-HBV_low | 5 | 6 | 16 | 0.931 | 1 | ns | ns |
| EndothelialCells | AAV-HBV_high | AAV-HBV_low | 5 | 6 | 9 | 0.329 | 1 | ns | ns |
| Granulocytes | AAV-HBV_high | AAV-HBV_low | 5 | 6 | 18 | 0.662 | 1 | ns | ns |
| Hepatocytes | AAV-HBV_high | AAV-HBV_low | 5 | 6 | 9 | 0.329 | 1 | ns | ns |
| KupfferCells | AAV-HBV_high | AAV-HBV_low | 5 | 6 | 16 | 0.931 | 1 | ns | ns |
| Monocytes | AAV-HBV_high | AAV-HBV_low | 5 | 6 | 24 | 0.126 | 1 | ns | ns |
| Neutrophils | AAV-HBV_high | AAV-HBV_low | 5 | 6 | 29 | 0.009 | 0.121 | ** | ns |
| NK | AAV-HBV_high | AAV-HBV_low | 5 | 6 | 29 | 0.009 | 0.121 | ** | ns |
| PlasmaCells | AAV-HBV_high | AAV-HBV_low | 5 | 6 | 13 | 0.792 | 1 | ns | ns |
| Proliferating | AAV-HBV_high | AAV-HBV_low | 5 | 6 | 4 | 0.052 | 0.727 | ns | ns |
| StromalCells | AAV-HBV_high | AAV-HBV_low | 5 | 6 | 9 | 0.329 | 1 | ns | ns |

*AAV-HBV low vs naive*

| **CellType** | **group1** | **group2** | **n1** | **n2** | **statistic** | **p** | **p.adj** | **p.signif** | **p.adj.signif** |
| --- | --- | --- | --- | --- | --- | --- | --- | --- | --- |
| B | AAV-HBV_low | naive | 6 | 5 | 9 | 0.329 | 1 | ns | ns |
| CD3+ | AAV-HBV_low | naive | 6 | 5 | 18 | 0.662 | 1 | ns | ns |
| Cholangiocytes | AAV-HBV_low | naive | 6 | 5 | 18 | 0.662 | 1 | ns | ns |
| DC | AAV-HBV_low | naive | 6 | 5 | 20 | 0.429 | 1 | ns | ns |
| EndothelialCells | AAV-HBV_low | naive | 6 | 5 | 23 | 0.177 | 1 | ns | ns |
| Granulocytes | AAV-HBV_low | naive | 6 | 5 | 6 | 0.126 | 1 | ns | ns |
| Hepatocytes | AAV-HBV_low | naive | 6 | 5 | 9 | 0.329 | 1 | ns | ns |
| KupfferCells | AAV-HBV_low | naive | 6 | 5 | 21 | 0.329 | 1 | ns | ns |
| Monocytes | AAV-HBV_low | naive | 6 | 5 | 18 | 0.662 | 1 | ns | ns |
| Neutrophils | AAV-HBV_low | naive | 6 | 5 | 10 | 0.429 | 1 | ns | ns |
| NK | AAV-HBV_low | naive | 6 | 5 | 13 | 0.792 | 1 | ns | ns |
| PlasmaCells | AAV-HBV_low | naive | 6 | 5 | 16 | 0.931 | 1 | ns | ns |
| Proliferating | AAV-HBV_low | naive | 6 | 5 | 24 | 0.126 | 1 | ns | ns |
| StromalCells | AAV-HBV_low | naive | 6 | 5 | 17 | 0.792 | 1 | ns | ns |

*AAV-HBV high vs naive*

| **CellType** | **group1** | **group2** | **n1** | **n2** | **statistic** | **p** | **p.adj** | **p.signif** | **p.adj.signif** |
| --- | --- | --- | --- | --- | --- | --- | --- | --- | --- |
| B | AAV-HBV_high | naive | 5 | 5 | 10 | 0.69 | 1 | ns | ns |
| CD3+ | AAV-HBV_high | naive | 5 | 5 | 17 | 0.421 | 1 | ns | ns |
| Cholangiocytes | AAV-HBV_high | naive | 5 | 5 | 18 | 0.31 | 1 | ns | ns |
| DC | AAV-HBV_high | naive | 5 | 5 | 20 | 0.151 | 1 | ns | ns |
| EndothelialCells | AAV-HBV_high | naive | 5 | 5 | 17 | 0.421 | 1 | ns | ns |
| Granulocytes | AAV-HBV_high | naive | 5 | 5 | 5 | 0.151 | 1 | ns | ns |
| Hepatocytes | AAV-HBV_high | naive | 5 | 5 | 6 | 0.222 | 1 | ns | ns |
| KupfferCells | AAV-HBV_high | naive | 5 | 5 | 21 | 0.095 | 1 | ns | ns |
| Monocytes | AAV-HBV_high | naive | 5 | 5 | 22 | 0.056 | 0.778 | ns | ns |
| Neutrophils | AAV-HBV_high | naive | 5 | 5 | 23 | 0.032 | 0.444 | * | ns |
| NK | AAV-HBV_high | naive | 5 | 5 | 23 | 0.032 | 0.444 | * | ns |
| PlasmaCells | AAV-HBV_high | naive | 5 | 5 | 13 | 1 | 1 | ns | ns |
| Proliferating | AAV-HBV_high | naive | 5 | 5 | 10 | 0.69 | 1 | ns | ns |
| StromalCells | AAV-HBV_high | naive | 5 | 5 | 15 | 0.69 | 1 | ns | ns |
